# Selective photocatalytic oxidation of aromatic alcohols to aldehydes with air by magnetic WO_3_ZnO/Fe_3_O_4_. *In situ* photochemical synthesis of 2-substituted benzimidazoles†

**DOI:** 10.1039/d0ra08403d

**Published:** 2020-11-09

**Authors:** Bozhi Li, Reza Tayebee, Effat Esmaeili, Mina S. Namaghi, Behrooz Maleki

**Affiliations:** Department of Food Science and Engineering, Jinzhou Medical University Jinzhou China rtayebee@hsu.ac.ir; Department of Chemistry, School of Sciences, Hakim Sabzevari University Sabzevar 96179-76487 Iran; Department of Chemistry, Payame Noor University (PNU) Tehran 19395-4697 Iran

## Abstract

Recently, visible light-driven organic photochemical synthesis has been a pioneering field of interest from academic and industrial associations due to its unique features of green and sustainable chemistry. Herein, WO_3_ZnO/Fe_3_O_4_ was synthesized, characterized, and used as an efficient magnetic photocatalyst in the preparation of a range of 2-substituted benzimidazoles *via* the condensation of benzyl alcohol and *o*-phenylenediamine in ethanol at room temperature for the first time. The key feature of this work is focused on the *in situ* photocatalytic oxidation of benzyl alcohols to benzaldehydes under atmospheric air and in the absence of any further oxidant. This new heterogeneous nanophotocatalyst was characterized *via* XRD, FT-IR, VSM and SEM. Short reaction time, cost-effectiveness, broad substrate scope, easy work-up by an external magnet, and excellent product yield are the major advantages of the present methodology. A number of effective experimental parameters were also fully investigated to clear broadness and generality of the protocol.

## Abstract 2

WO_3_ZnO/Fe_3_O_4_ is used as a magnetic photocatalyst in the preparation of 2-substituted benzimidazoles in EtOH at RT. The key feature is the *in situ* photocatalytic oxidation of benzyl alcohols to benzaldehydes under atmospheric air in the absence of further oxidant.

## Graphical abstract

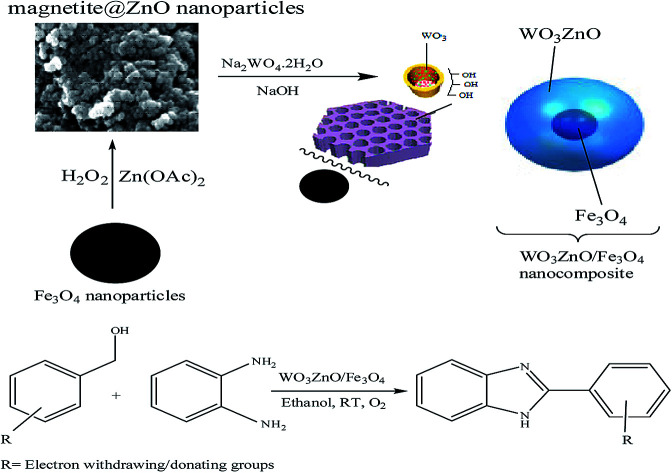

## 1. Introduction

Recently, green energy issues have attracted considerable attention to develop newer effective sustainable energy resources and environmentally benign routes to achieve important synthetic pharmaceuticals broadly used in the human life. The visible region of the solar energy (∼43%) is the most useful alternative to perform many green organic photochemical reactions under catalytic conditions due to its sustainable and abundant nature.^1^ Therefore, a great deal of concern is devoted to the photocatalytic degradation of organic wastes in water resources, efficient photocatalytic oxyfunctionalization of organic compounds and selective organic transformations under ambient environmentally friendly conditions, in particular with atmospheric air as a natural oxidant, under solar light.^2–6^ Among various organic transformations, the selective photochemical oxidation of alcohols to carbonyl compounds is of prime importance due to its significant role in the synthesis of a variety of fine chemicals, confectionaries, fragrances, and pharmaceutical industries.^7–17^

A photocatalyst is capable of absorbing light and producing electron–hole pairs to force chemical reactions.^18^ Among the various photocatalysts, a great deal of attention has been paid to semiconducting metal oxides because of their compatible band gap in the visible region. Usually, the absorption of a photon by a semiconductor with an energy equal to or greater than the band gap energy can transfer an electron from the valence band to the conduction band, leaving a hole in the conduction band with a high oxidation capacity. The generated hole can absorb electrons from the hydroxyl ions in water to generate highly unstable and reactive ·OH. Therefore, the excited electron can also react with the oxygen gas to produce an O_2_^−^ radical-anion. The created electron–hole pair can follow different routes to react with the organic materials adsorbed on the surface of the photocatalyst. Notable features such as optimum band gap, wide surface area, good stability and reusability and suitable morphology are intended from an effective photocatalyst. In addition, the performed photocatalyst should be fabricated under environmentally friendly and cost-effective conditions.^18–20^ Furthermore, the photocatalyst concentration, reactant concentration, type of the chemical transformation, temperature, kind and intensity of the light source can directly affect the performance and effectiveness of a photochemical reaction.^21,22^ Semiconducting metal oxides such as zinc, titanium, tungsten, vanadium, and cerium oxides are the most familiar photocatalysts, which have been used extensively in electronic and chemical technology, hydrogen production, storage, and environmental remediation because of their suitable electronic structure, charge transfer characteristics, and light absorption properties.^18,22–24^

Among nitrogen-containing heteroaromatics, benzimidazoles are vital core materials to generate different and significant drugs. They exhibit various biological activities such as antiviral,^25^ anti-inflammatory,^26^ antibacterial,^27^ antiparasitic,^28^ analgesic,^29^ antihypertensive,^30^ antituberculosis,^31^ and antiprotozoal effects.^32^ Considering such a wide range of biological functionalities, extensive efforts have been made to the progress of efficient strategies for the synthesis of various 2-substituted benzimidazoles. Condensation of 2-aminothiophenol, *o*-phenylenediamine, and 2-aminophenol with aromatic aldehydes under acidic conditions is the well-known method to prepare these drugs^33^ by the mediation of various catalysts such as magnetic catalysts,^34^ porous catalysts,^35^ metal–organic framework catalysts,^36^ ionic liquid catalysts,^37^ heavy metal catalysts,^38^ graphene oxides^39^ and so on. Moreover, quinoxalines are also important heteroaromatic compounds that have drawn extensive attention for use in many pharmaceuticals,^40,41^ various biofunctional molecules,^42^ semiconductor materials^43^ and organic synthons.^44^

**Scheme 1:**
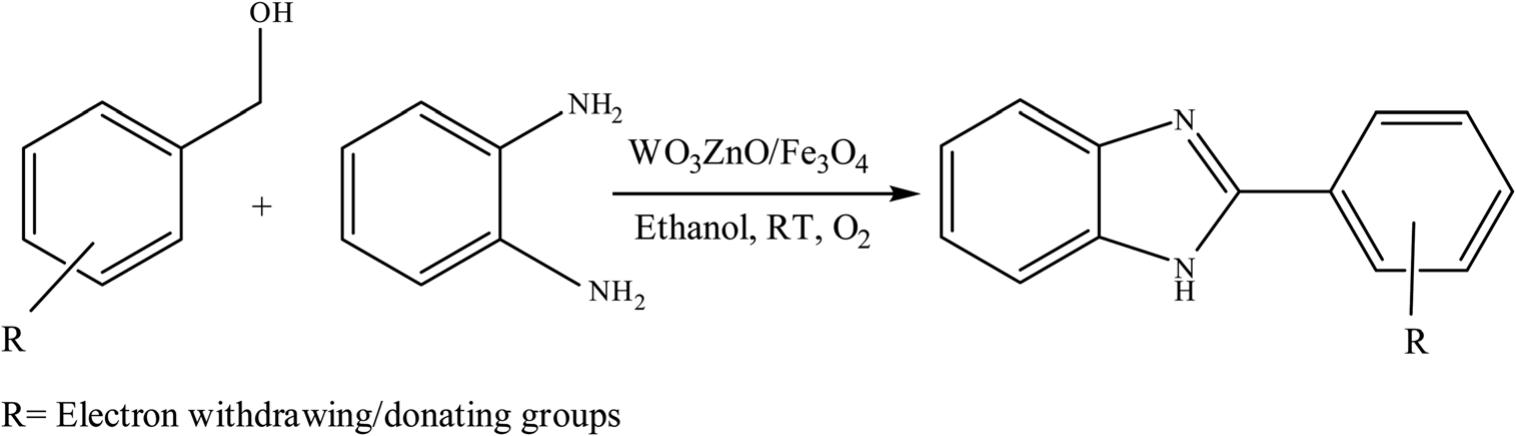
General route for the preparation of different 2-substituted benzimidazoles.

To the best of our knowledge, WO_3_ZnO/Fe_3_O_4_ was prepared and investigated for the first time as an ideal magnetic nanophotocatalyst in the synthesis of 2-substituted benzimidazoles *via* the reaction of *ortho*-substituted anilines with various substituted benzyl alcohols in the presence of air as a natural oxidant under the irradiation of a high-pressure Hg lamp (Scheme 1). The impacts of various experimental conditions such as solvent type, reactant concentration, temperature, contact time, and source of illumination on the efficacy of the photocatalytic synthesis were also monitored. Furthermore, we have used different benzyl alcohols with different electron-rich and electron-deficient groups in the condensation with anilines.

## 2. Experimental section

### 2.1. Materials and methods

Zinc chloride, sodium hydroxide, zinc acetate, sodium tungstate, benzyl alcohols, and other starting materials and solvents were purchased from Merck and used without further treatment. A high-pressure mercury vapor lamp (NAVIFLUX, 400 W, Berlin, NARVA) with a lamp operating current of 3.25 A and a nominal voltage of 230 V was used for the photocatalytic experiments. The light intensity of the UVC lamp was equal to 1020 μW cm^−2^ as measured using a Spectroline model DRC-100X digital radiometer combined with a DIX-365 radiation sensor (Shokofan Tosee, Iran). ^1^H and ^13^C-NMR spectra were recorded using a 300 MHz Bruker AVANCE III spectrometer in DMSO-d_6_ using TMS as the internal reference. Melting points were determined in open capillary tubes using a Stuart BI Branstead Electrothermal cat. no. IA9200 apparatus and uncorrected. The morphology of the synthesized nanomaterials was studied using a Tescan 3Mira 3-XMU field emission scanning electron microscope (FESEM). Fourier transform infrared (FT-IR) spectroscopy was performed using a Shimadzu 8700 Fourier transform spectrophotometer in the range of 400 to 4000 cm^−1^ with KBr pellets. UV-visible spectra were recorded using a Photonix UV-visible array spectrophotometer. X-ray diffraction (XRD) patterns were acquired using an Xpert MPD diffractometer with Cu K_α_ radiation at 30 mA and 40 keV and a scanning rate of 3° min^−1^ in the 2*θ* domain from 5° to 80°. The room-temperature magnetic measurements were carried out using a Vibrating Sample Magnetometer model of Magnetic Daghigh Kavir, MDKB.

### 2.2. Preparation of Fe_3_O_4_ nanoparticles by a chemical co-precipitation method

Fe_3_O_4_ magnetic nanoparticles were prepared according to the previously reported procedure.^45^ FeCl_3_·6H_2_O (10.3 g) and FeCl_2_·4H_2_O (3.9 g) were dissolved in 100 mL deionized water, purged with N_2_ for 15 min, and heated to 85 °C. Thereafter, 15 mL of NH_4_OH solution (32%) was added drop-wise as well. The precipitated solid material was separated using an external magnet after 15 min and washed thoroughly with a NaCl solution (0.1 M, 3 × 10 mL). Finally, the obtained dark powder was dried overnight in a vacuum oven at 80 °C for 3 h.

### 2.3. Preparation of Fe_3_O_4_ nanoparticles *via* bioreduction of *S. cumini* seed extract (Fe_3_O_4_ SMNT)

Fe_3_O_4_ SMNT was prepared by an easy and environmentally friendly method. First, 2.20 g of FeCl_3_·6H_2_O and 6.60 g of sodium acetate were dissolved in 50 mL of freshly prepared *S. cumini* seed extract. The resulting solution contained polysaccharides and other biomolecules, and the mixture was agitated at 67 °C for 2 h. After 2 h, the resulting solution became a black homogeneous dispersion, indicating generation of Fe_3_O_4_ SMNT. The colloidal solution was cooled to 25 °C and the black precipitate was separated using an external magnetic field, washed three times thoroughly with ethanol and dried overnight in vacuum at 90 °C overnight.

### 2.4. Preparation of zinc oxide nanoparticles

In a typical route, 12.6 g of hydrated zinc acetate was dissolved in 450 mL of double-distilled water under stirring until it was completely dissolved. Thereafter, the solution was heated to 50 °C, and 500 mL of absolute alcohol was slowly added under stirring. Then, 6 mL of H_2_O_2_ (47%) was added drop-wise and mixed using a magnetic stirrer until the solution becomes clear. This solution was treated for 24 h and, then, dried at 80 °C for several hours to obtain the white nanopowder of zinc oxide. The attained zinc oxide was washed with double-distilled water to completely remove contaminants. After that, ZnO nanoparticles were dried at 80 °C to attain complete conversion and growth of the crystals.^46^

### 2.5. Preparation of WO_3_ZnO and WO_3_ZnO/Fe_3_O_4_ nanoparticles

First, 30 mL of NaOH (4 M) was added drop-wise into a 20 mL solution of zinc acetate (1 M) and Na_2_WO_4_·2H_2_O (2 mL, 0.5 M). Then, the obtained suspension was maintained at room temperature under mild continuous stirring for 3 h to form the precursor. After that, the obtained suspension was subsequently transferred into a 250 mL ground-glass-stopped conical flask and aged at 95 °C for 10 h. Finally, the generated precipitate was collected, washed with distilled water, and air-dried at room temperature. To fabricate WO_3_ZnO/Fe_3_O_4_, the preparation method for WO_3_ZnO was repeated as described, but, in the presence of the as-synthesized Fe_3_O_4_ nanoparticles (0.3 g).

### 2.6. Preparation of melamine/H_3_PW_12_O_40_ nanoparticles

First, 0.1 g of melamine was added to 10 mL of chloroform and left on the stirrer for 1 h to dissolve. During this process, pure concentrated sulfuric acid was added drop-wise to the solution to obtain an oily solution. Thereafter, 0.1 g of H_3_PW_12_O_40_ was dissolved in 5 mL of deionized water and slowly added to the above oily mixture. The final mixture was stirred at room temperature to yield a white powder. After stirring for 2 h, the white solid powdered material was filtered, washed several times with water and dried under an open-air autoclave.

### 2.7. General experimental procedure for the synthesis of 2-substituted benzimidazoles

A mixture of benzyl alcohol (1 mmol), *o*-phenylenediamine (1 mmol), and WO_3_ZnO/Fe_3_O_4_ nanophotocatalyst (0.01 g) was taken in 10 mL ethanol at room temperature. Then, the stirring reaction mixture was exposed to the HP mercury light irradiation under mild air bubbling (1 mL min^−1^), and progress of the reaction was monitored by TLC. After completion of the reaction, the magnetic nanophotocatalyst was separated easily from the homogeneous reaction mixture using an external magnet. Finally, the yellow product appeared by adding ice-water to this solution. The precipitated product was filtered and re-crystallized in ethanol to bring further purified products. For the sake of completeness, the NMR characterization data for 2-substituted benzimidazoles (3a–m) are provided in Table 3.

## 3. Results and discussion

### 3.1. Characterization and physicochemical properties of WO_3_ZnO/Fe_3_O_4_ nanoparticles

The physicochemical and morphological properties of the supported WO_3_ZnO/Fe_3_O_4_ nanophotocatalyst were explored using the most informative techniques such as FESEM, FT-IR, VSM, and XRD.

#### 3.1.1. FESEM

**Fig. 1:**
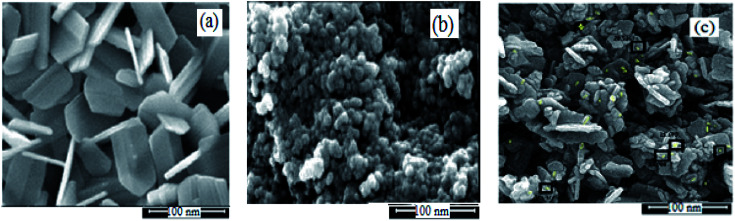
FESEM images of WO_3_ (a), ZnO (b), and WO_3_ZnO/Fe_3_O_4_ (c) nanoparticles.

**Fig. 2:**
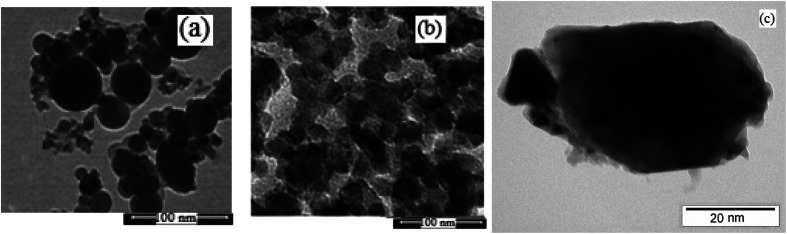
TEM images of WO_3_ (a), ZnO (b), and WO_3_ZnO/Fe_3_O_4_ (c) nanocomposites.

Electron microscopy is field emission electron microscopy that enables surface photography and morphology of the nanoparticles with a magnification of about 500 000 times and with a resolution of about 1–20 nm. Field-emission scanning electron microscopy was used to monitor the morphology and particle distribution in WO_3_ (Fig. 1a), ZnO (Fig. 1b), and WO_3_ZnO/Fe_3_O_4_ (Fig. 1c). From FE-SEM images, it can be seen that the prepared nanocomposite had a uniform surface morphology with a concomitant irregular distribution. The FESEM image of WO_3_ZnO/Fe_3_O_4_ nanoparticles showed random and irregular distribution of nanoparticles. The surface of the material also showed layered and plate-like nanoparticles. In addition, the TEM images of WO_3_ (Fig. 2a), ZnO (Fig. 2b), and WO_3_ZnO/Fe_3_O_4_ (Fig. 2c) are also provided. It can be found that the surface of the WO_3_–ZnO nanocomposite constructed revealed layered-shape particles with an average thickness of about 27 nm and with effectively enhanced surface area, providing superior photocatalytic performance.

#### 3.1.2. FT-IR spectroscopy

**Fig. 3:**
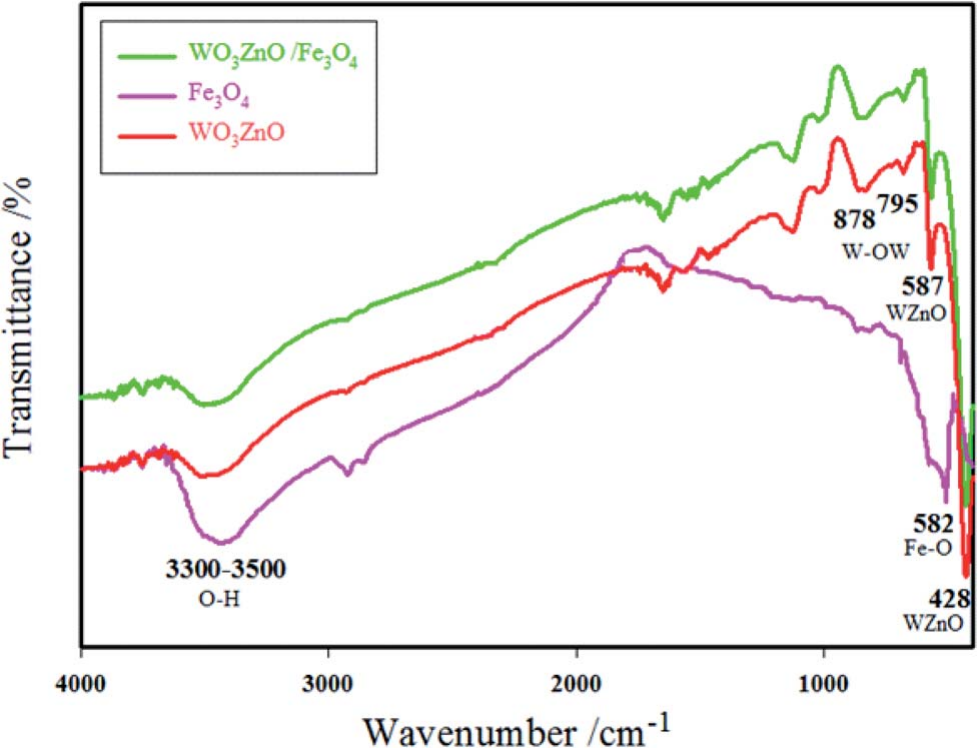
FT-IR spectra of Fe_3_O_4_, WO_3_ZnO, and WO_3_ZnO/Fe_3_O_4_.

Fig. 3 shows the FT-IR spectra of Fe_3_O_4_, WO_3_ZnO, and WO_3_ZnO/Fe_3_O_4_ in the range of 400–4000 cm^−1^. The peak absorbed at 587 cm^−1^ belonged to the Fe–O vibration and the broad absorption peak at 3300–3500 cm^−1^ was due to the surface hydroxide ions.^47^ The absorption band at 428 and 587 cm^−1^ were due to the ZnO moiety, whereas a weak and nearly wide band at 883 cm^−1^ would belong to WO_3_.^48,49^

#### 3.1.3. XRD

**Fig. 4:**
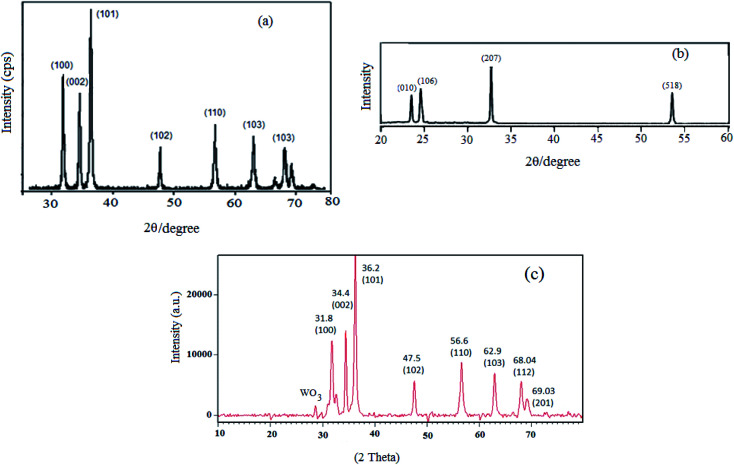
Wide-angle XRD patterns of ZnO (a), WO_3_ (b), and WO_3_ZnO/Fe_3_O_4_ (c).

The XRD method is the most common technique to identify the crystal structure and crystallinity of a powdered material. Therefore, the relative amount of crystal phases along with the average particle size of WO_3_ZnO/Fe_3_O_4_ can be easily determined by this technique. The phase analysis of ZnO, WO_3_, and WO_3_ZnO (WZnO) was performed by XRD (Fig. 4). The XRD patterns of ZnO (Fig. 4a) and WO_3_ (Fig. 4b) were in good agreement with the literature. The peaks observed in the 2*θ* range of 31.8, 34.4, 36.2, 47.5, 56.6, 62.9, 68.04 and 69.03 were, respectively, assigned to the crystalline planes of (100), (002), (101), (102), (110), (103), (112) and (201) due to the presence of ZnO, which matched well with the JCPDS file no. 89-0510.^50^ The XRD pattern of WO_3_ZnO/Fe_3_O_4_ nanoparticles was almost comparable to that of pure ZnO nanoparticles.^51^ The intensity of all diffraction patterns was not altered, which indicated that the ZnO crystallinity did not change after doping with WO_3_.^52^ Noteworthy, the weak diffraction peak with the 2*θ* value of 28.2 can be indexed to the hexagonal phase of WO_3_ (JCPDS no. 33-1387).^53^ The low intensity of WO_3_ diffraction would be due to the low amount and amorphous phase of tungsten oxide among ZnO nanoparticles.

#### 3.1.4. X-ray photoelectron emission spectroscopy (XPS) of WZnO

**Fig. 5:**
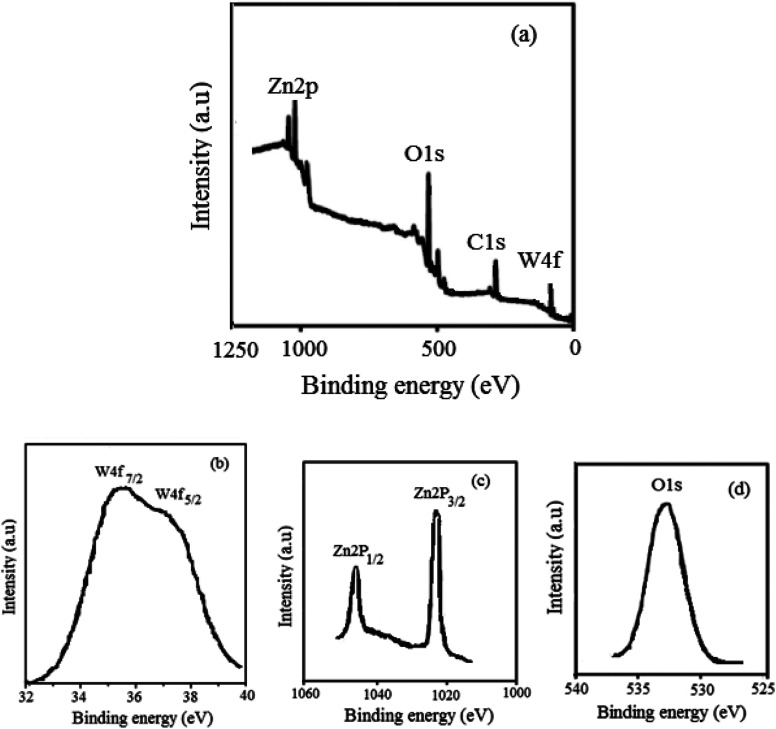
XPS survey spectrum of WZnO (a), W 4f (b), Zn 2p (c), and O 1s (d) in WO_3_ZnO.

The superficial chemical conformation and valance states of W, Zn, and O in WZnO were disclosed by XPS. The spectra were calibrated according to the C 1s line with the corresponding binding energy of 286.5 eV. The XPS spectra survey in Fig. 5a confirmed no other elements rather than Zn, O, W, and C. The C 1s peak originated from the carbon tape used in the instrument. The high-resolution deconvoluted XPS spectra of Zn 2p, W 4f, and O 1s of WZnO are shown in Fig. 5b–d. The spin–orbit components of 2p_3/2_ and 2p_1/2_ due to Zn 2p peaks were observed at 1021.9 and 1044.9 V, respectively, confirming that Zn exists in the form of Zn^2+^ ions. The O 1s spectrum shows two peaks at 532.6 and 533.7 eV, which are attributed to the vacancy oxygen atoms of ZnO and surface-chemisorbed hydroxyl groups, respectively.^54,55^ Moreover, the binding energy values of 36.2 and 37.8 eV were due to the spin–orbit components of 4f_7/2_ and 4f_5/2_ for W 4f peaks, respectively (Fig. 5b), confirming the W^6+^ form in the nanocomposite.

#### 3.1.5. VSM

**Fig. 6:**
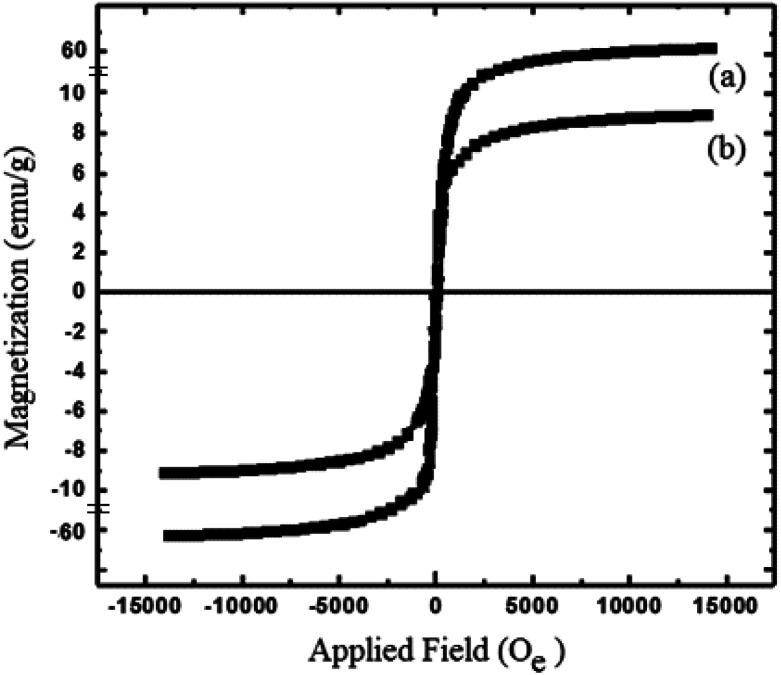
VSM magnetization curves of Fe_3_O_4_ (a) and WO_3_ZnO/Fe_3_O_4_ (b) nanoparticles.

The magnetic property of WO_3_ZnO/Fe_3_O was compared with bare Fe_3_O_4_ by vibrating sample magnetometry (VSM) at 25 °C (Fig. 6). The saturation magnetization of the bare magnetic and WO_3_ZnO/Fe_3_O_4_ nanoparticles was ∼61 and 9.0 emu g^−1^, respectively. These results confirmed the formation of the well-defined crystalline structure for the magnetite nanoparticles. This study confirmed that a shell of WO_3_ZnO significantly decreased the saturation magnetization. However, the saturation magnetization of WO_3_ZnO/Fe_3_O_4_ was high enough to guarantee easy separation of it using an external strong magnet from the reaction mixture.

### 3.2. Photocatalytic preparation of 2-substituted benzimidazoles and studying different reaction parameters

#### 3.2.1. Studying the photocatalytic activity of some simple or mixed metal oxides, semi-conductors, and fused N-heterocycles

**Fig. 7:**
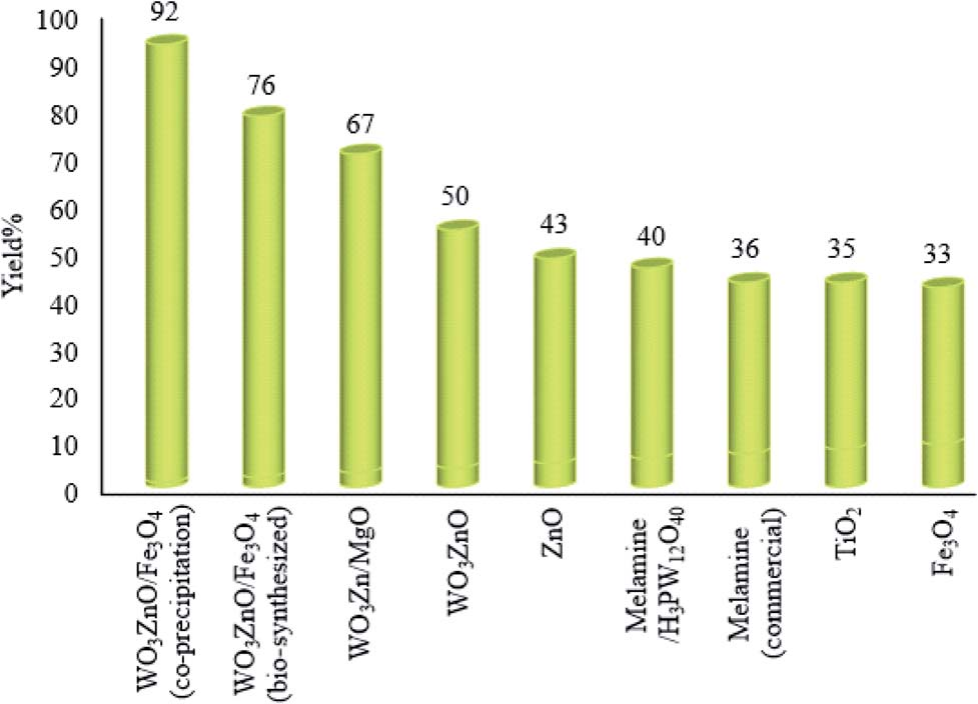
Photocatalytic activity of some simple or mixed metal oxides in the synthesis of 2-substituted benzimidazoles. *o*-Phenylenediamine (1 mmol), benzyl alcohol (1 mmol), and 2 mg of each photocatalyst were mixed in 10 mL ethanol under the irradiation of a high-pressure Hg lamp at 25 °C for 3 h. Yield% refers to the isolated yield.

In these experiments, a mixture of *o*-phenylenediamine (1 mmol), benzyl alcohol (1 mmol), and 2 mg of each photocatalyst was prepared in 10 mL ethanol and illuminated with a high-pressure Hg lamp for 3 h. This study was planned to achieve the best photocatalyst in the desired condensation reaction (Fig. 7). According to the attained findings, WO_3_ZnO/Fe_3_O_4_, prepared by the co-precipitation method, behaved significantly much better that the bio-synthesized counterpart wads found as the best photocatalyst to attain 92% yield after 3 h. After that, the mixed metal oxide WO_3_Zn/MgO showed good photocatalytic activity and provided 67% yield during the same time. Noteworthy, doping of WO_3_ZnO with Mg (∼10 wt%) was constructive and increased the yield% from 50 to 67%, respectively. Comparison of WO_3_ZnO with ZnO clearly confirmed that doping with WO_3_ is constructive and improves the photocatalytic efficacy of the semi-conductor in the desired photochemical condensation reaction. It seems that TiO_2_ is just a little less reactive than ZnO in this reaction. Interestingly, Fe_3_O_4_ is an effective photocatalyst in the synthesis of 2-substituted benzimidazoles, and it attains 33% yield. This finding promoted us to use magnetite as a core in the final nanophotocatalyst WO_3_ZnO/Fe_3_O_4_ for the two reasons of increasing the photocatalytic efficacy and simplifying the separation of the nanophotocatalyst using an external magnetic field. Since fused nitrogen-containing materials such as graphitic carbon nitride and melem have been used as photocatalysts in the degradation of colored organic wastes, we tested for the first time commercial melamine as the trimer of cyanamide by acting as a photocatalyst and found that it can produce 36% yield after 3 h. Although this material did not show high photocatalytic activity, this finding is important because melamine is a major byproduct of most petrochemical industries. Finally, to improve the photocatalytic activity of melamine, phosphotungstic acid (H_3_PW_12_O_40_) was selected as a strong superacid to activate the lone pair of nitrogen and form a kind of ionic liquid. However, the prepared material showed only a little progress in the photocatalytic activity and led to 40% yield after 3 h.

#### 3.2.2. Effect of the reaction time on the condensation reaction

**Fig. 8:**
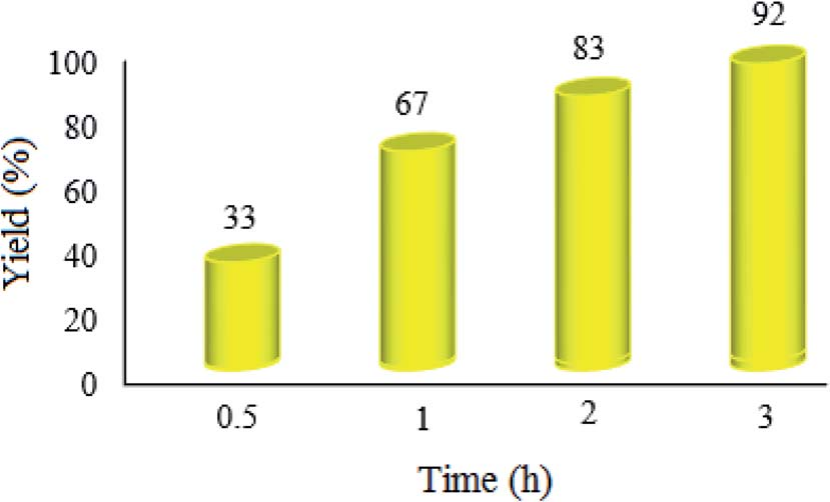
Effect of the reaction time on the condensation reaction. The reaction conditions are the same as described for Fig. 7; 20 mg of WO_3_ZnO/Fe_3_O_4_ was used in all cases.

Initial attempts to optimize the reaction time in the synthesis of 2-substituted benzimidazoles were made using benzyl alcohol as the model substrate in the presence of WO_3_ZnO/Fe_3_O_4_ under the reaction conditions mentioned in Fig. 7 description. This experiment confirmed that 2.5 h duration is adequate to acquire 89% yield (Fig. 8).

#### 3.2.3. Studying the impact of catalyst amount on the photocatalytic efficacy

**Fig. 9:**
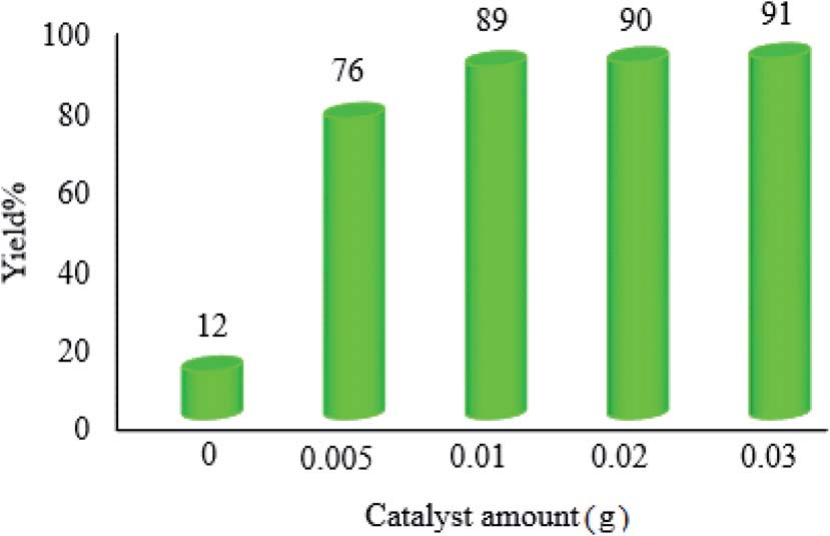
Effect of the catalyst amount on the photocatalytic efficacy of WO_3_ZnO/Fe_3_O_4_. The reaction conditions are the same as described for Fig. 7. Reactions were performed for 2.5 h. Yield% was increased to 18 and 31% in the absence of photocatalyst after 4 and 24 h, respectively.

In this case, we carried out some reactions with different amounts of the photocatalyst in order to optimize the exact quantity of WO_3_ZnO/Fe_3_O_4_. As shown in Fig. 9, 0.01 g of the photocatalyst was appropriate for the present pathway under the standard reaction conditions. However, a further increment in the amount of catalyst affected neither the yield% nor the reaction time.

#### 3.2.4. Studying the effect of benzyl alcohol : *o*-phenylenediamine molar ratio

**Fig. 10:**
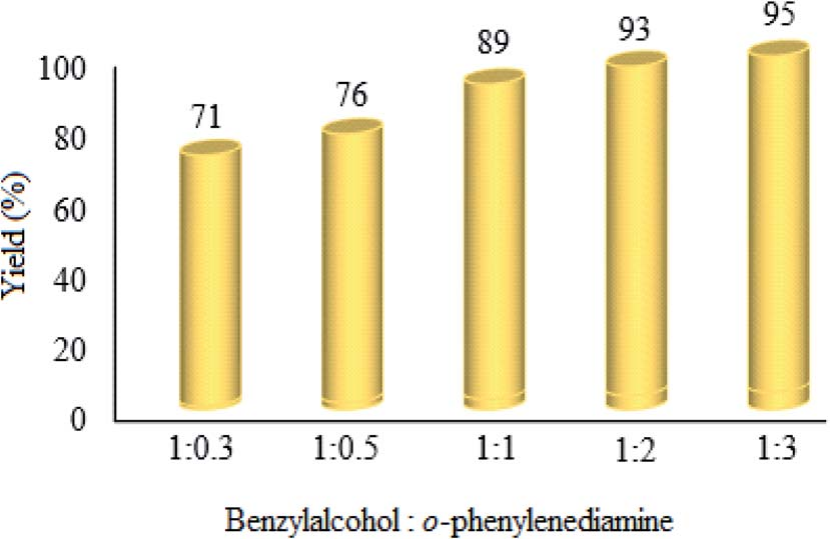
Effect of benzyl alcohol : *o*-phenylenediamine ratio on the photocatalytic efficacy of WO_3_ZnO/Fe_3_O_4_. Here, 10 mg of WO_3_ZnO/Fe_3_O_4_ was used in all cases. Reactions were performed for 2.5 h.

The effect of changes in the ratio of raw materials was investigated, and it was found that the product yield increases with the increase in ratio of benzyl alcohol : *o*-phenylenediamine (Fig. 10). Clearly, the yield% was enhanced with the mentioned ratio. However, the 1 : 1 ratio was chosen as the optimum, because no significant improvement was attained at higher ratios.

#### 3.2.5. Studying different irradiation sources on the photocatalytic condensation reaction

In another important experiment, the effect of different light sources was investigated, and it was found that different lights have a profound effect on the photocatalytic efficacy of the synthetic route. The attained results showed that only 4% yield was gained under dark conditions, whereas the product yield was improved to 17% by means of a 50 W white LED. A commercial xenon lamp (1000 W) yielded 38%; however, the best result (89%) was attained with UV-Vis irradiation under similar reaction conditions, as selected for the optimum (Table 1).

**Table 1:** Studying different irradiation sources on the photocatalytic efficacy of WO_3_ZnO/Fe_3_O_4_

Catalyst	Light source	Yield%
WO_3_ZnO/Fe_3_O_4_ chemical	High pressure Hg lamp	89
WO_3_ZnO/Fe_3_O_4_ chemical	Xenon lamp	38
WO_3_ZnO/Fe_3_O_4_ chemical	White LED	17
WO_3_ZnO/Fe_3_O_4_ chemical	No light	4
WO_3_ZnO/Fe_3_O_4_ chemical	Sunlight	45 (8 h)

A mixture of benzyl alcohol (1 mmol), *o*-phenylenediamine (1 mmol), and WO_3_ZnO/Fe_3_O_4_ nanophotocatalyst (0.01 g) was taken in 10 mL ethanol at room temperature for 2.5 h. The stirring reaction mixture was exposed to the light source under mild air bubbling (1 mL min^−1^) and progress of the reaction was monitored by TLC.

#### 3.2.6. Synthesis of different 2-substituted benzimidazoles catalyzed by WO_3_ZnO/Fe_3_O_4_

By consideration of the optimized reaction conditions in hand, we investigated the scope and generality of the process with respect to *o*-phenylenediamine and benzyl alcohol using some substituted benzyl alcohols (Table 2).^56^ Both electron-withdrawing and electron-donating groups on the phenyl ring of alcohol were used as substrates and gave the corresponding products in good to excellent yields with no significant effect on the efficiency of the transformation, although benzyl alcohols with electron-withdrawing substituents gave relatively better yields than those containing electron-donating substituents. Therefore, substituted benzyl alcohols with various functional groups such as NO_2_, Cl, and CH_3_ reacted smoothly to deliver the desired products.^56,57^

**Table 2:** Synthesis of different 2-substituted benzimidazoles catalyzed by WO_3_ZnO/Fe_3_O_4_

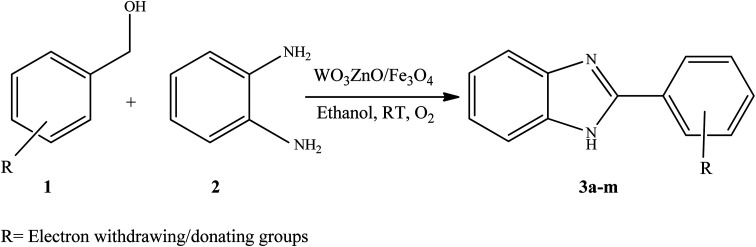				
Entry	Benzyl alcohols	Product	Yield (%)	Mp (°C)
1	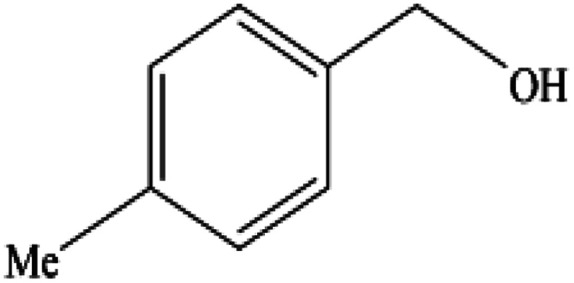	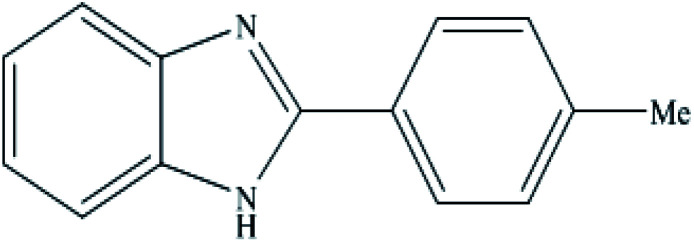	92	263–265
2	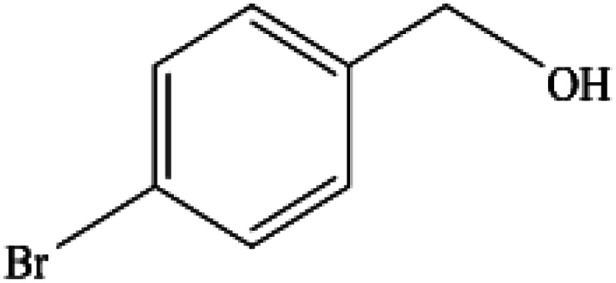	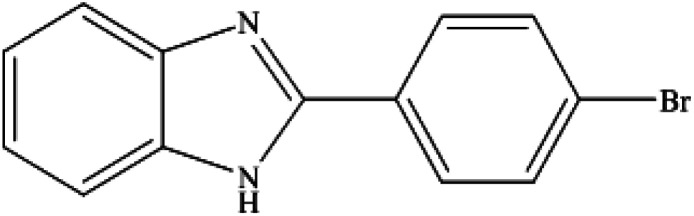	95	291–293
3	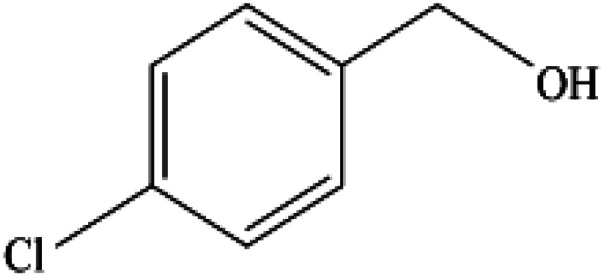	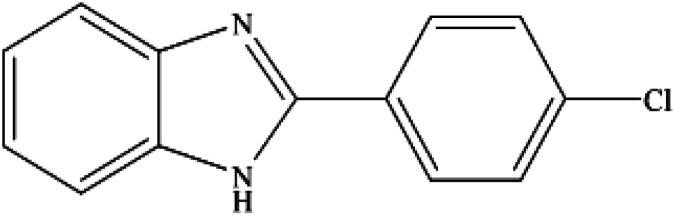	96	289–291
4	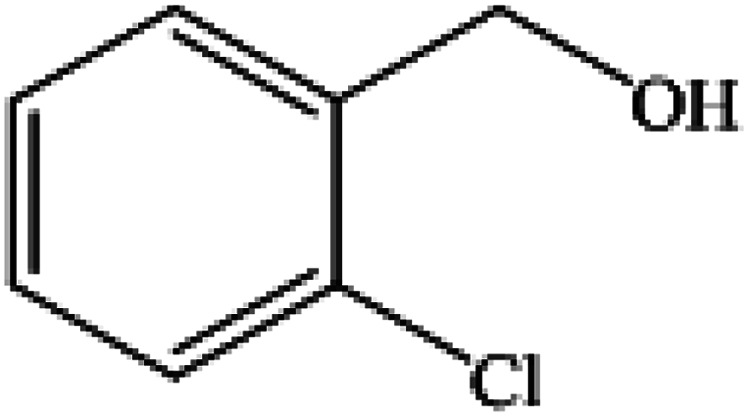	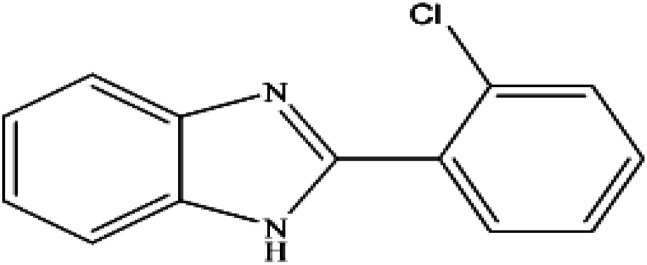	93	228–230
5	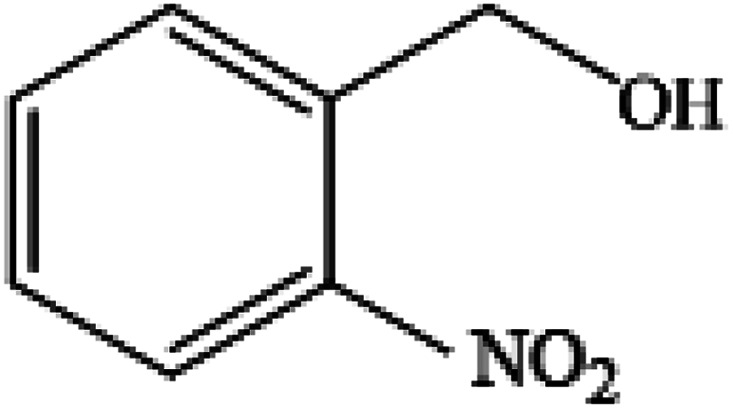	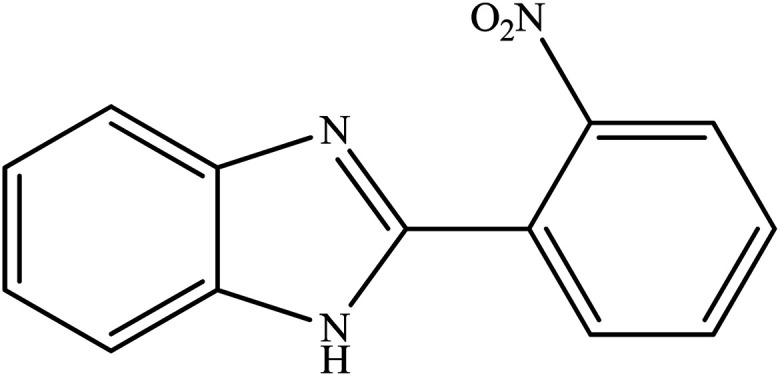	97	211–213
6	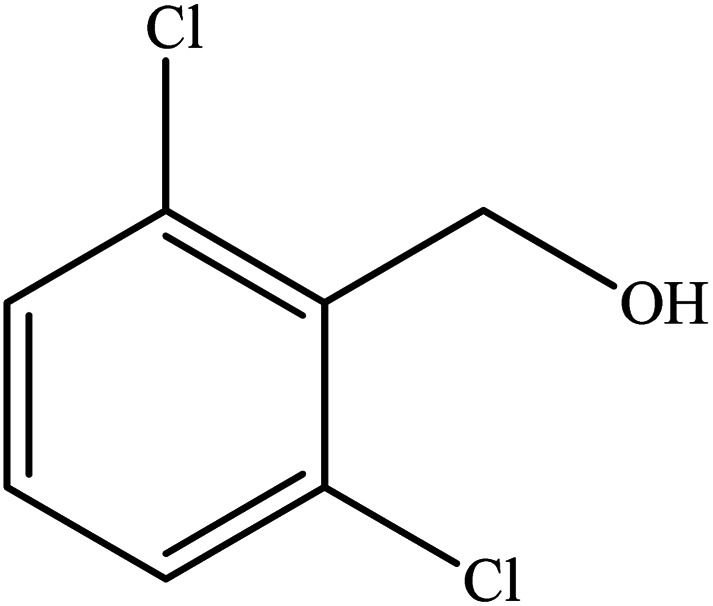	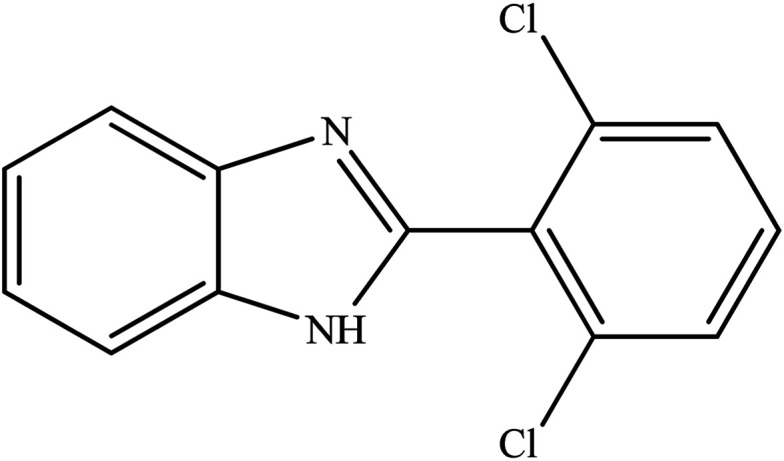	98	274–276
7	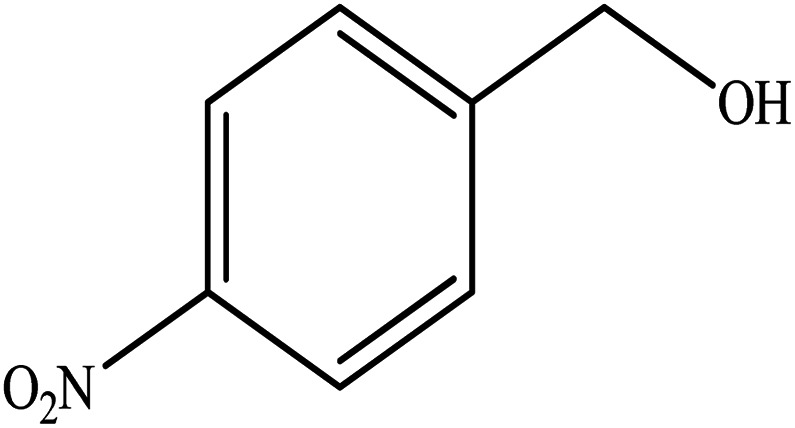	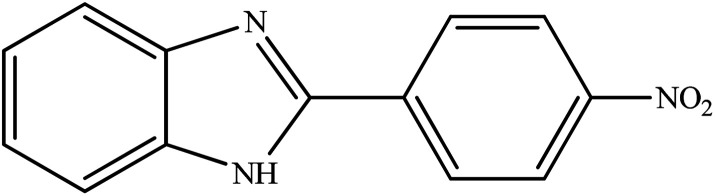	98	310–312
8	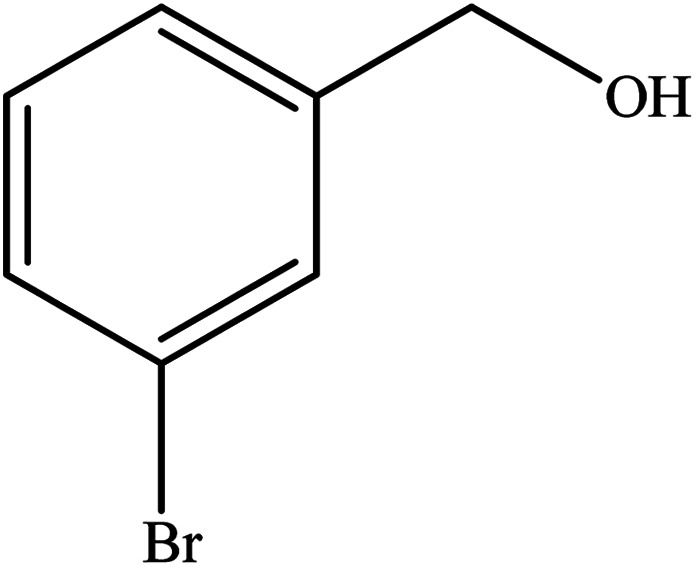	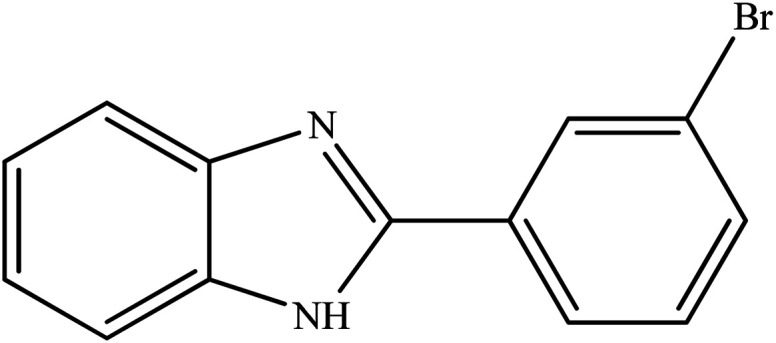	92	281–283
9	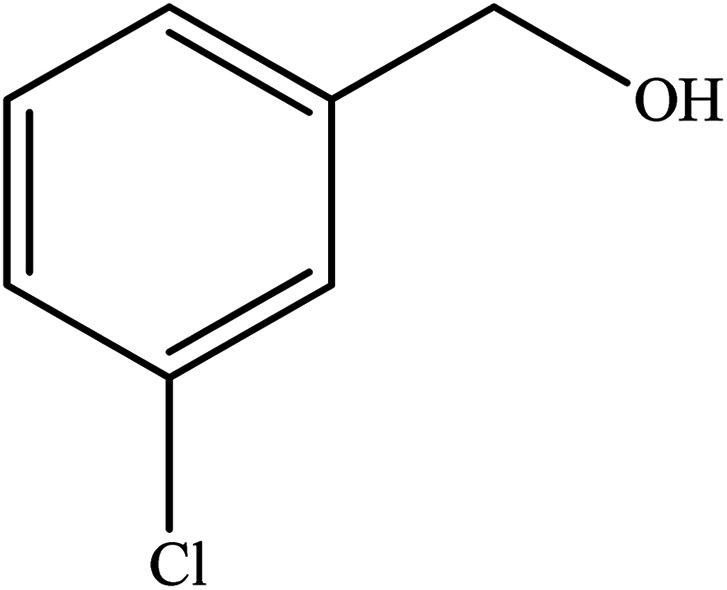	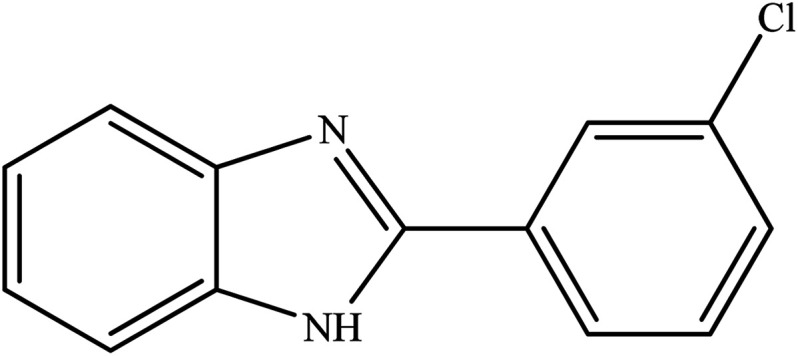	93	255–257
10	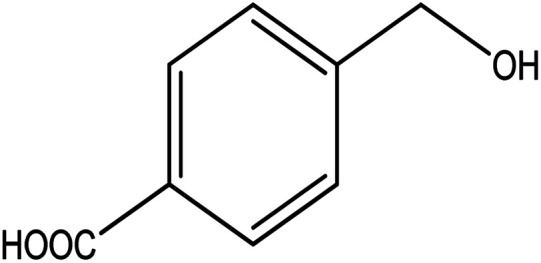	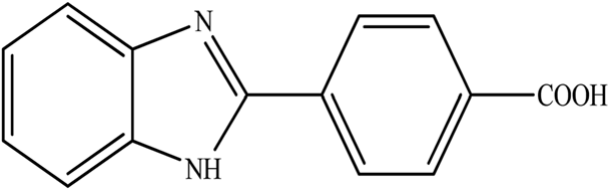	98	>300
11	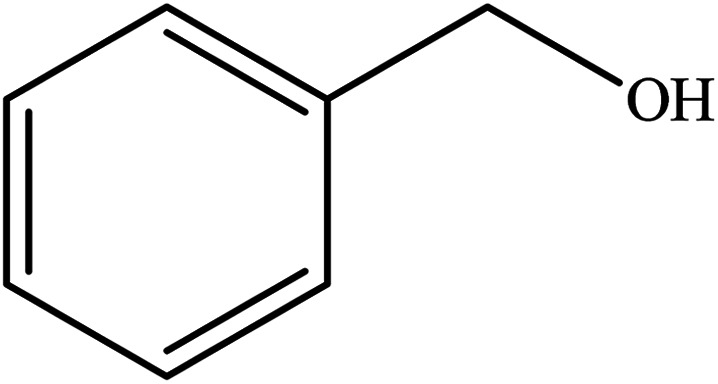	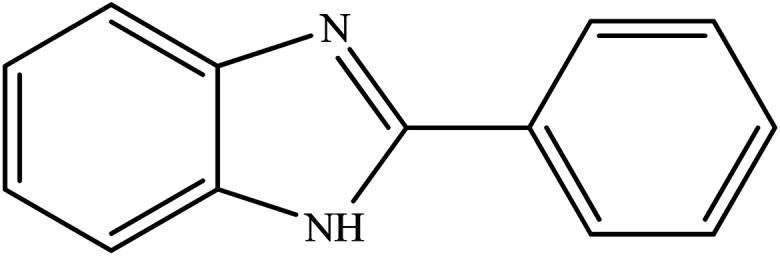	90	291–293
12	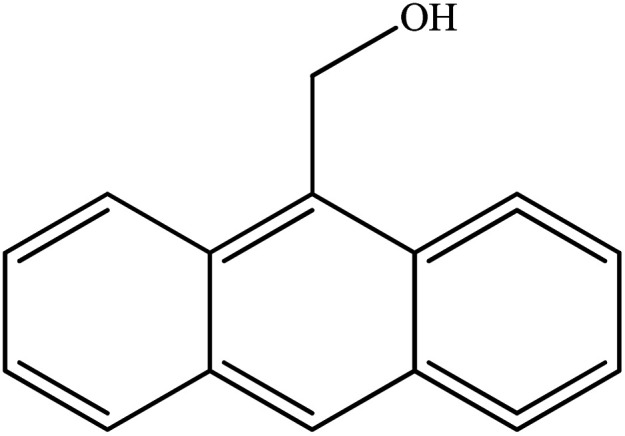	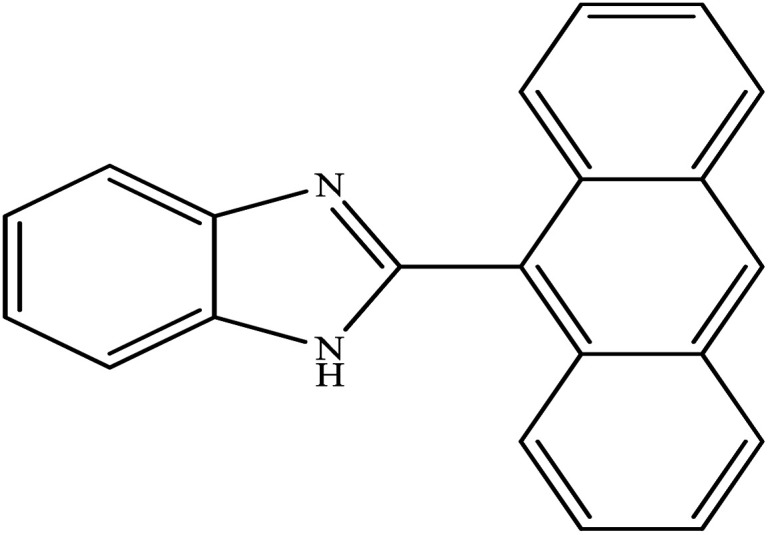	90	>330
13	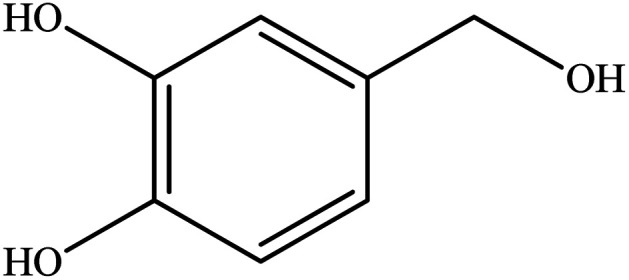	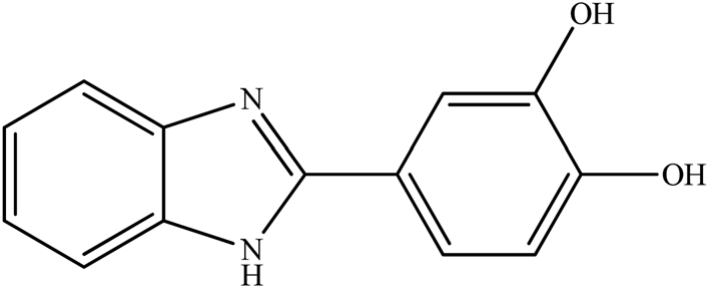	88	270–272

**Table 3:** NMR spectral data of the prepared 2-substituted benzimidazoles

Product	NMR spectral data of 2-substituted benzimidazoles (3a–m)
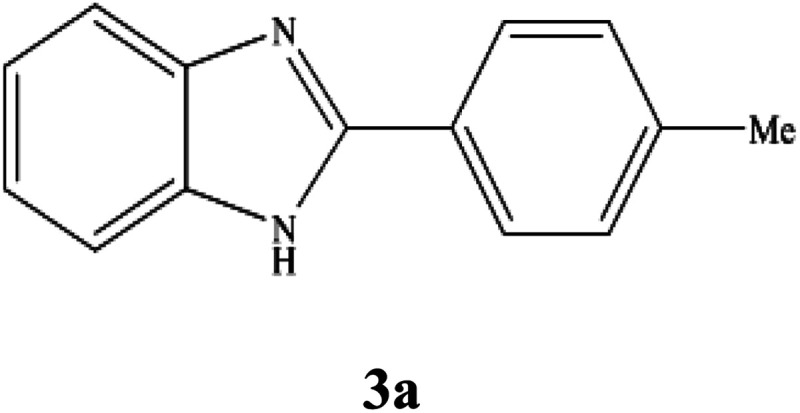	^1^H NMR (300 MHz, DMSO-d_6_); *δ* 2.39 (s, 3H), 7.20–7.22 (m, 2H), 7.36–7.39 (d, 2H), 7.55–7.66 (m, 2H), 8.09–8.11 (d, 2H), 12.85 (s, 1H, NH) ppm; ^13^C NMR (75 MHz, DMSO-d_6_); *δ* 21.44, 111.68, 119.18, 122.05, 122.77, 126.87, 127.95, 129.98, 135.45, 140.02, 144.20, 151.87 ppm
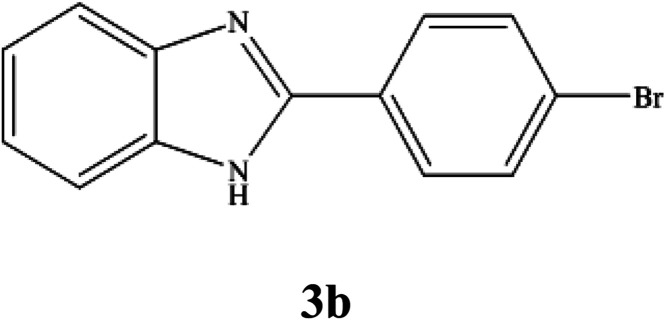	^1^H NMR (300 MHz, DMSO-d_6_); *δ* 7.23–7.26 (m, 2H), 7.55–7.64 (m, 4H), 8.17–8.20 (m, 1H), 8.26–8.28 (m, 1H), 12.77 (brs, 1H, NH) ppm; ^13^C NMR (75 MHz, DMSO-d_6_); *δ* 111.94, 119.45, 122.47, 123.14, 123.74, 128.85, 129.91, 132.43, 135.63, 144.25, 150.74 ppm
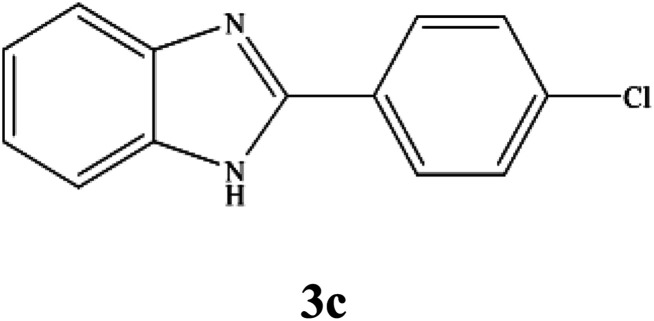	^1^H NMR (300 MHz, DMSO-d_6_); *δ* 7.20–7.28 (m, 2H), 7.55–7.57 (d, 2H), 7.64–7.71 (m, 3H), 8.19–8.23 (d, 2H), 13.00 (s, 1H, NH) ppm; ^13^C NMR (75 MHz, DMSO-d_6_); *δ* 111.89, 119.45, 122.31, 123.26, 128.61, 129.54, 129.55, 134.96, 135.50, 144.23, 150.63 ppm
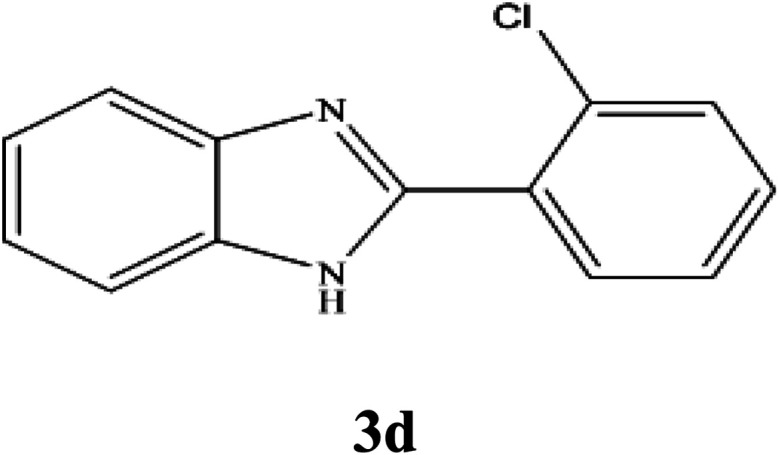	^1^H NMR (300 MHz, DMSO-d_6_); *δ* 7.34–7.36 (m, 2H), 7.42–7.45 (m, 2H), 7.51–7.54 (m, 1H), 7.72–7.74 (m, 2H), 8.42–8.45 (m, 1H), 10.45 (brs, 1H, NH) ppm
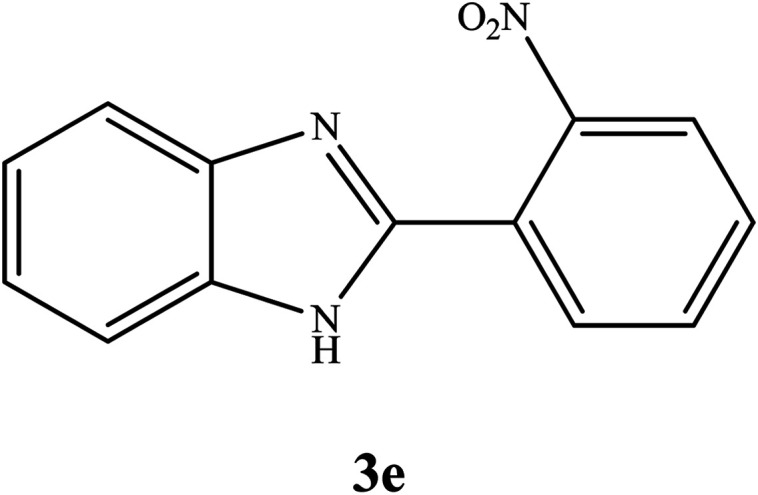	^1^H NMR (300 MHz, DMSO-d_6_); *δ* 7.25–7.30 (m, 2H), 7.59–7.61 (m, 1H), 7.68–7.70 (m, 1H), 7.75–7.80 (m, 1H), 7.86–7.91 (t, 1H), 7.99–8.07 (m, 2H), 13.09 (s, 1H, NH) ppm; ^13^C NMR (75 MHz, DMSO-d_6_); *δ* 112.14, 122.37, 123.56, 124.73, 124.77, 131.36, 131.41, 131.44, 133.11, 135.10, 144.09, 147.79, 149.44 ppm
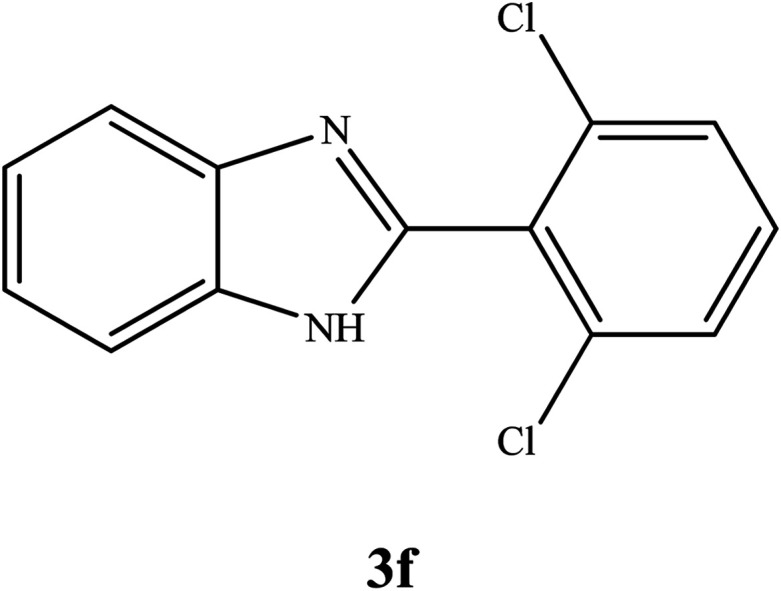	^1^H NMR (300 MHz, DMSO-d_6_); *δ* 7.24–7.26 (m, 2H), 7.58–7.59 (m, 2H), 7.61–7.67 (m, 1H), 8.17–8.20 (m, 1H), 8.26–8.27 (m, 1H), 13.03 (brs, 1H, NH) ppm; ^13^C NMR (75 MHz, DMSO-d_6_); *δ* 112.05, 119.77, 122.04, 123.24, 128.80, 128.82, 131.10, 132.85, 134.56, 135.54, 143.69, 143.76, 147.17 ppm
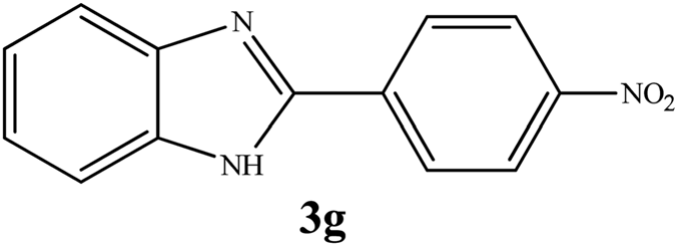	^1^H NMR (300 MHz, DMSO-d_6_); *δ* 7.28–7.29 (d, 2H), 7.60–7.72 (d, 2H), 8.36–8.44 (m, 4H), 13.27 (s, 1H, NH) ppm; ^13^C NMR (75 MHz, DMSO-d_6_); *δ* 112.24, 119.92, 122.74, 124.01, 124.67, 127.80, 135.67, 136.49, 144.30, 148.20, 149.45 ppm
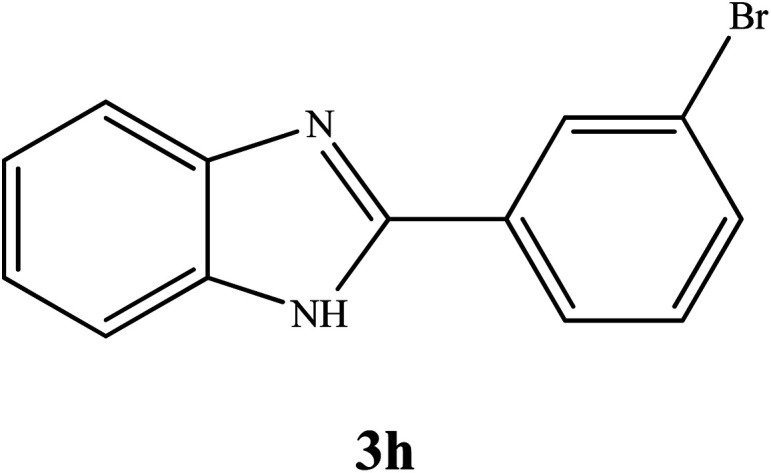	^1^H NMR (300 MHz, DMSO-d_6_); *δ* 7.25–7.26 (m, 2H), 7.51–7.56 (m, 2H), 7.70–7.72 (m, 2H), 8.22–8.25 (d, 1H), 8.41 (s, 1H), 13.18 (brs, 1H, NH) ppm; ^13^C NMR (75 MHz, DMSO-d_6_); *δ* 111.98, 119.53, 122.42, 122.72, 123.36, 123.70, 125.88, 129.39, 131.64, 132.89, 135.46, 144.10, 150.09 ppm
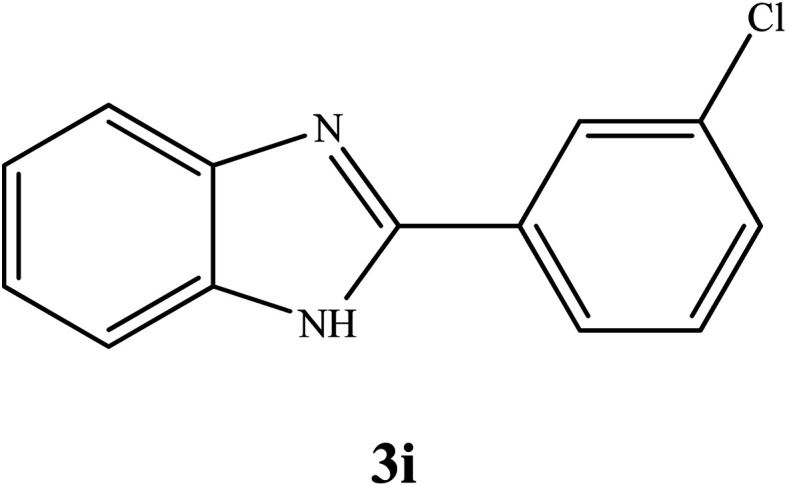	^1^H NMR (300 MHz, DMSO-d_6_); *δ* 7.24–7.26 (m, 2H), 7.58–7.59 (m, 2H), 7.61–7.64 (m, 1H), 7.69–7.70 (m, 1H), 8.17–8.20 (m, 1H), 8.26–8.27 (m, 1H), 13.03 (brs, 1H, NH) ppm; ^13^C NMR (75 MHz, DMSO-d_6_); *δ* 112.00, 119.54, 122.40, 123.39, 125.51, 126.51, 130.00, 131.41, 132.69, 134.23, 135.43, 144.13, 150.20 ppm
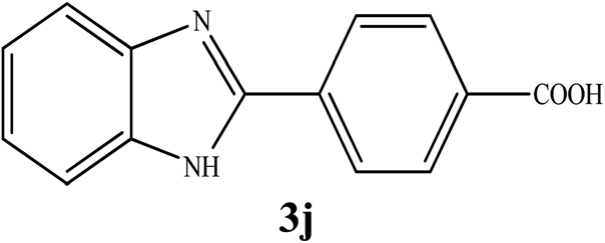	^1^H NMR (300 MHz, DMSO-d_6_); *δ* 7.24–7.29 (m, 2H), 7.64–7.66 (m, 2H), 8.12–8.15 (d, 2H), 8.31–8.34 (d, 2H), 13.18 (brs, 2H, NH, OH) ppm; ^13^C NMR (75 MHz, DMSO-d_6_); *δ* 123.02, 126.95, 130.41, 132.05, 134.43, 150.65, 167.37 ppm
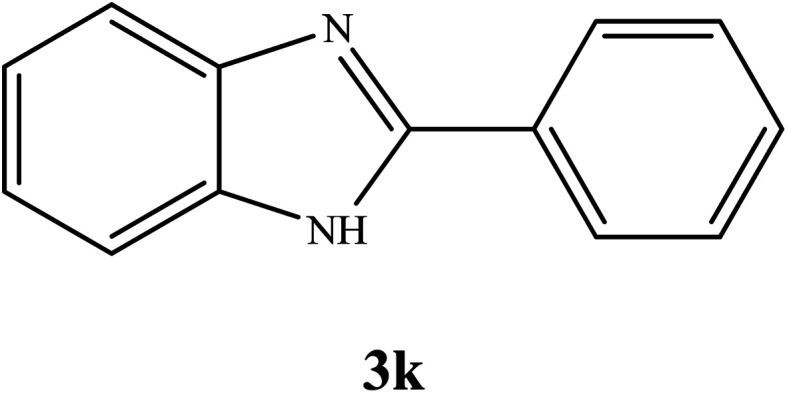	^1^H NMR (300 MHz, DMSO-d_6_); *δ* 7.24–7.25 (m, 3H), 7.56–7.58 (m, 2H), 7.62–7.64 (m, 2H), 8.22–8.23 (d, 2H), 13.10 (brs, 1H, NH) ppm; ^13^C NMR (75 MHz, DMSO-d_6_); *δ* 115.68, 122.68, 126.58, 126.98, 129.26, 129.43, 129.52, 130.41, 137.37, 143.03, 151.60 ppm
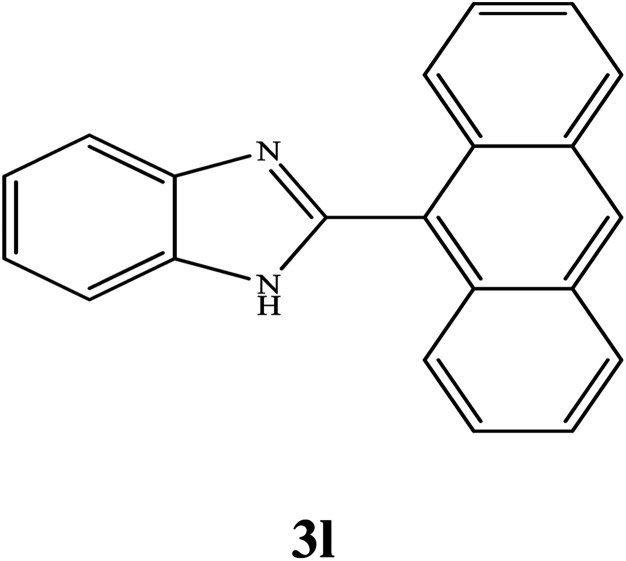	^1^H NMR (300 MHz, DMSO-d_6_); *δ* 6.76–6.81 (m, 1H), 6.96–6.99 (m, 1H), 7.15–7.20 (m, 1H), 7.42–7.45 (m, 1H), 7.54–7.60 (m, 4H), 8.09–8.11 (m, 2H), 8.63 (s, 1H), 8.78–8.81 (m, 2H), 9.79 (s, 1H, NH) ppm; ^13^C NMR (75 MHz, DMSO-d_6_); *δ* 115.46, 117.20, 117.99, 118.01, 121.23, 122.78, 125.39, 125.95, 127.64, 128.31, 128.49, 128.99, 129.39, 130.49, 131.21, 131.37, 135.36, 137.38, 144.35, 145.89, 156.58 ppm
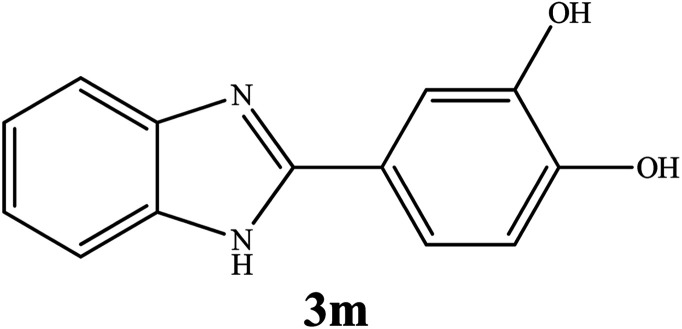	^1^H NMR (300 MHz, DMSO-d_6_); *δ* 7.24 (s, 2H), 7.62–7.70 (m, 4H), 8.22–7.25 (m, 2H), 13.02 (s, 1H, NH) ppm; ^13^C NMR (75 MHz, DMSO-d_6_); *δ* 111.89, 119.45, 122.31, 123.22, 128.45, 128.62, 129.26, 129.51, 131.24, 134.94, 135.54, 144.26, 150.67 ppm

A mixture of aromatic alcohol (1 mmol), *o*-phenylenediamine (1 mmol), and WO_3_ZnO/Fe_3_O_4_ nanophotocatalyst (0.01 g) was reacted in 10 mL ethanol at room temperature for 2.5 h. The stirring reaction mixture was exposed to the light source under mild air bubbling (1 mL min^−1^) and progress of the reaction was monitored by TLC. Yield% refers to the isolated product.

#### 3.2.7. Hot filtration test

A hot filtration test was arranged to ensure that the photocatalytic activity originates from the whole WO_3_ZnO/Fe_3_O_4_ and not from the species, which may be released to the reaction mixture. For this purpose, 10 mg of WO_3_ZnO/Fe_3_O_4_ was added to a mixture of benzyl alcohol and *o*-phenylenediamine in ethanol under the standard reaction conditions and the reaction was started as explained in the Experimental section for 2.5 h. At this part, the product yield of 47% was obtained after 45 min. Thereafter, the photocatalyst was separated using an external magnet and the reaction was continued for 150 min in the absence of photocatalysts. No more than 2% was added to the last yield and, eventually, 49% product was obtained in this experiment. This experiment obviously confirmed that the nanophotocatalyst retained its heterogeneous nature and none of the fragments had been leached to ethanol as a strong polar solvent.

#### 3.2.8. Recovery and reusability of WO_3_ZnO/Fe_3_O_4_

**Fig. 11:**
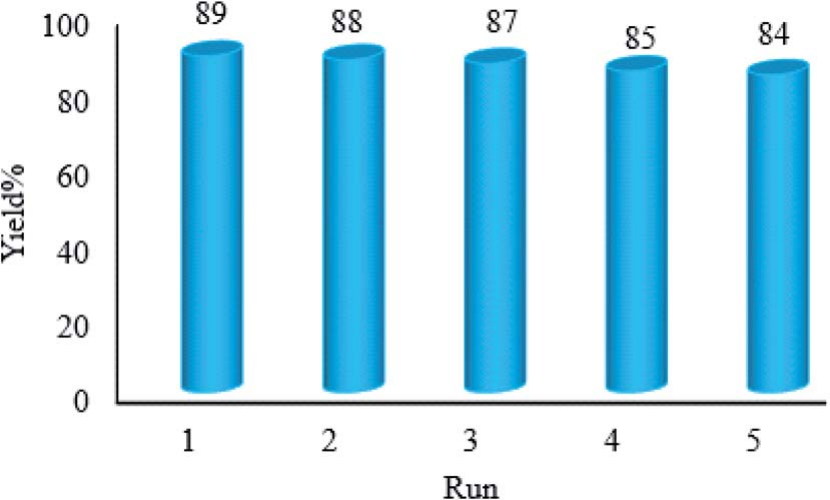
Studying recyclability of WO_3_ZnO/Fe_3_O_4_ under the optimized reaction conditions.

**Fig. 12:**
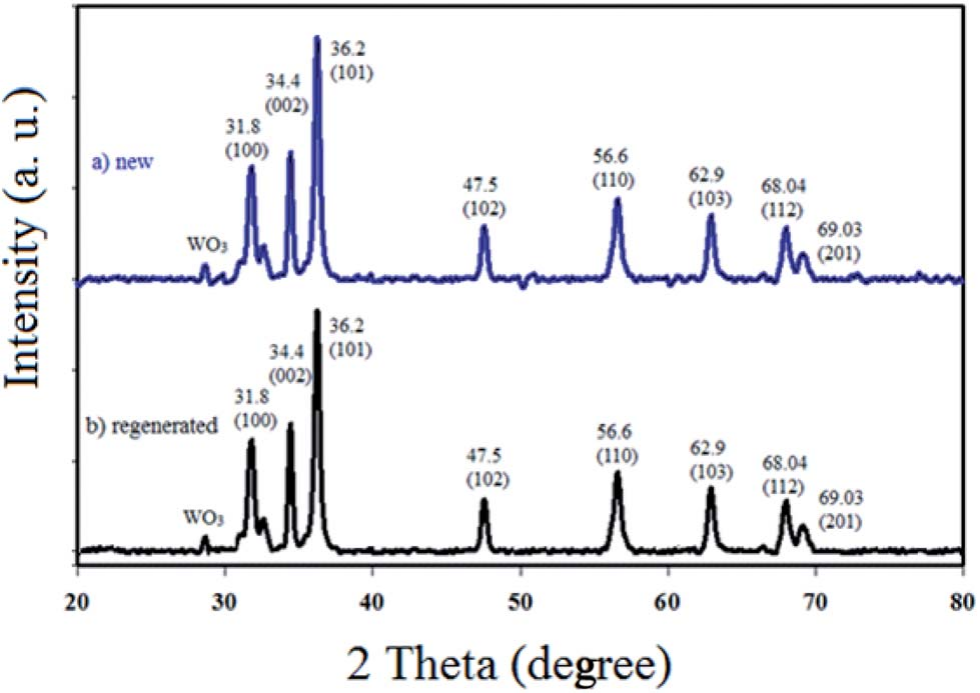
XRD patterns of the fresh (a) and final reused photocatalyst (b).

Recovery and reusability of the WO_3_ZnO/Fe_3_O_4_ photocatalyst are valuable properties from commercial and economic points of view, which must be evaluated in this project. Therefore, the reusability of this photocatalyst was investigated by repeatedly separating the catalyst from the reaction mixture and, then, reusing for over five consecutive runs. After each run, the catalyst was isolated from the reaction mixture using an external magnet, washed with copious amounts of ethanol and dried. Then, the recovered catalyst was applied for a subsequent new batch of the reaction (Fig. 11). This study clearly showed that the photocatalytic activity of WO_3_ZnO/Fe_3_O_4_ was preserved and no significant decrease was observed during reusing experiments. Furthermore, the XRD pattern of the final reused catalyst was compared to the fresh one and no significant change was observed (Fig. 12). This study clearly confirmed stability of the photocatalyst after reusing experiments.

#### 3.2.9. Proposed reaction pathway

**Scheme 2:**
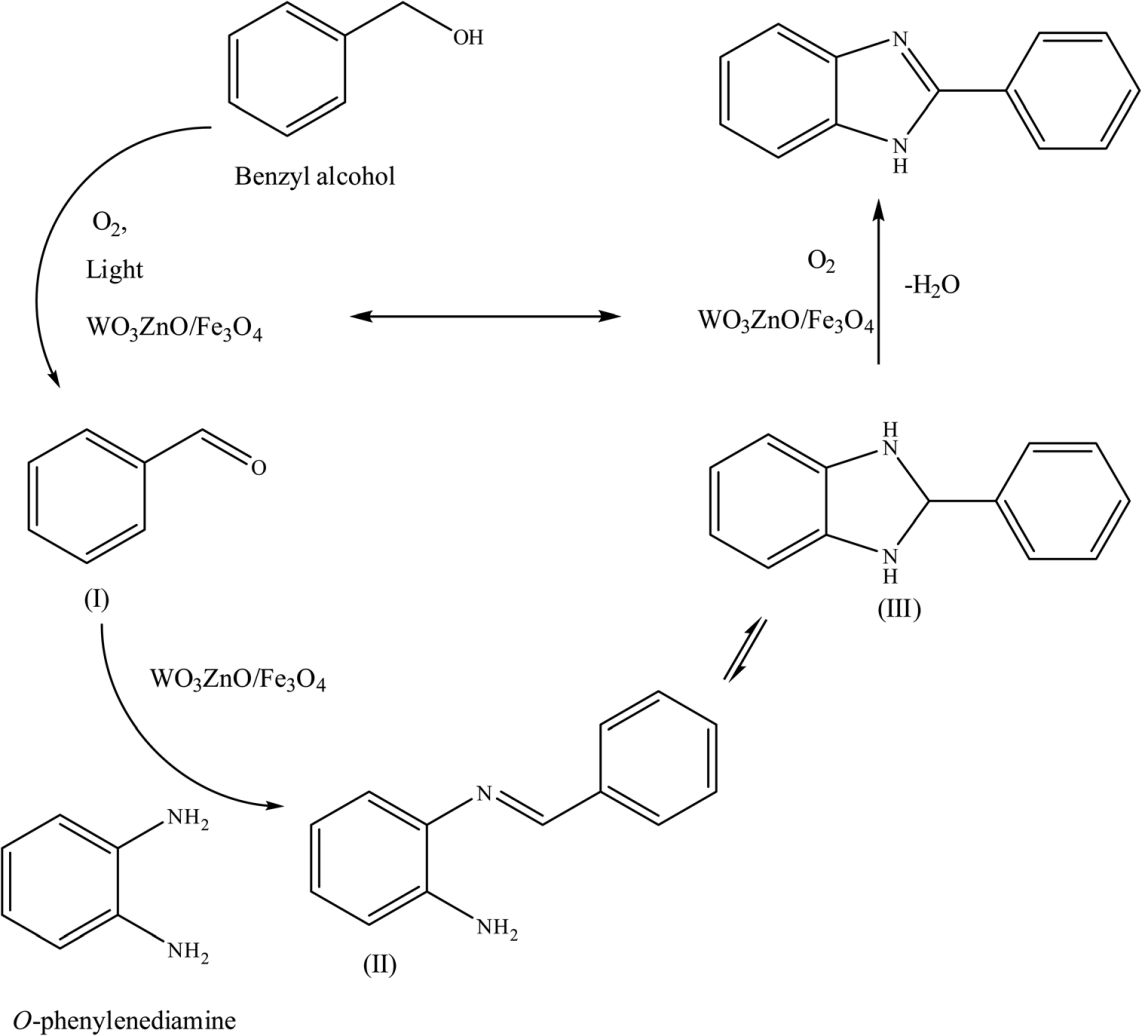
Plausible mechanism for the synthesis of different 2-substituted benzimidazoles catalyzed by WO_3_ZnO/Fe_3_O_4_.

A plausible reaction pathway is disclosed for this green and practical methodology, as described in Scheme 2. Initially, the benzyl alcohol substrate can be catalytically oxidized with air in the presence of WO_3_ZnO/Fe_3_O_4_ to form benzaldehyde (I). Then, phenylenediamine reacts with the *in situ* generated aldehyde to produce an imine intermediate (II), with concomitant release of a water molecule. The imine intermediate is in equilibrium with the corresponding dihydrobenzimidazole (III), and subsequently, it can be catalytically dehydrogenated in the presence of air and by the mediation of the prepared nanocatalyst to form the desired final benzimidazole product.^58^

## 4. Conclusions

We have developed a straightforward photochemical synthesis strategy of 2-substituted benzimidazoles from the condensation of benzyl alcohols with *o*-phenylenediamine in the presence of WO_3_ZnO/Fe_3_O_4_ in ethanol. This new heterogeneous nanophotocatalyst was characterized by XRD, FT-IR, VSM and SEM. It is noteworthy that all produced benzimidazoles were obtained in good to excellent yields. The used photocatalyst shows excellent recovery and reusability without any significant loss of the photocatalytic activity. The key feature of this work is the *in situ* photocatalytic oxidation of a range of benzyl alcohols to benzaldehydes under atmospheric air and in the absence of any further oxidant. In addition, the present protocol is also viable to convert secondary aromatic alcohols to quinoxalines. Moreover, ethanol is used as a green solvent instead of the common harmful solvents for the synthesis of 2-substituted benzimidazoles. This methodology has several benefits such as high photocatalytic activity, simple operation, high yield, and use of a minimum amount of the photocatalyst. To the best of our knowledge, this report is the first on the preparation of 2-substituted benzimidazoles under the photocatalytic conditions by means of a commercially available Hg lamp. More importantly, this photochemical reaction can be achieved under the irradiation of sunlight as a plentiful free source of solar light.

## Conflicts of interest

There are no conflicts to declare.

## Supplementary Material

RA-010-D0RA08403D-s001

RA-010-D0RA08403D-s002

RA-010-D0RA08403D-s003
