# Supplementary material for: Selective photocatalytic oxidation of aromatic alcohols to aldehydes with air by magnetic WO3ZnO/Fe3O4. In situ photochemical synthesis of 2-substituted benzimidazoles
Source: RSC Adv. 2020 Nov 9;10(67):40725–38. doi: 10.1039/d0ra08403d (PMC9057692; doi:10.1039/d0ra08403d)
Supplement: RA-010-D0RA08403D-s003 [file RA-010-D0RA08403D-s003.pdf]

Electronic Supplementary Material (ESI) for RSC Advances.
This journal is © The Royal Society of Chemistry 2020

Supporting Information

Selective photocatalytic oxidation of aromatic alcohols to aldehydes with air by magnetic WO_3ZnO/Fe_3O_4 . *In situ* photochemical synthesis of 2-substituted benzimidazoles

Bozhi Li[1], Reza Tayebee[*,2], Effat Esmaeili[3], Mina Shafiee[2], Behrooz Maleki[2]

[1]Department of Food Science and Engineering, Jinzhou Medical University, Jinzhou, China

[2]Department of Chemistry, School of Sciences, Hakim Sabzevari University, Sabzevar, 96179-76487, Iran

[3]Department of Chemistry, Payame Noor University (PNU), Tehran, 19395-4697, Iran

General

^{1}H- and ^{13}C-NMR spectra were recorded on a 300 MHz Bruker AVANCEC III spectrometer in DMSO-d_6 using TMS as the internal reference.

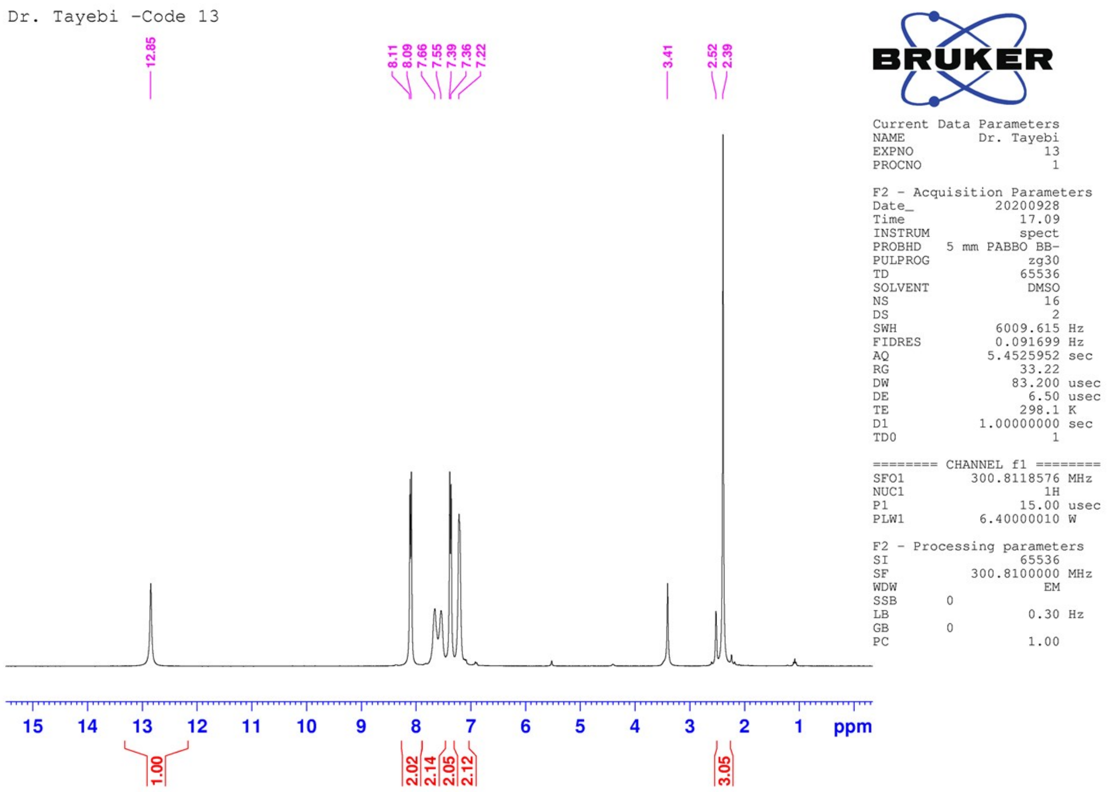

Fig. S1 ^{1}H-NMR Spectrum of 2-(4-methyl-phenyl)-benzimidazole (Table 2, entry 1).

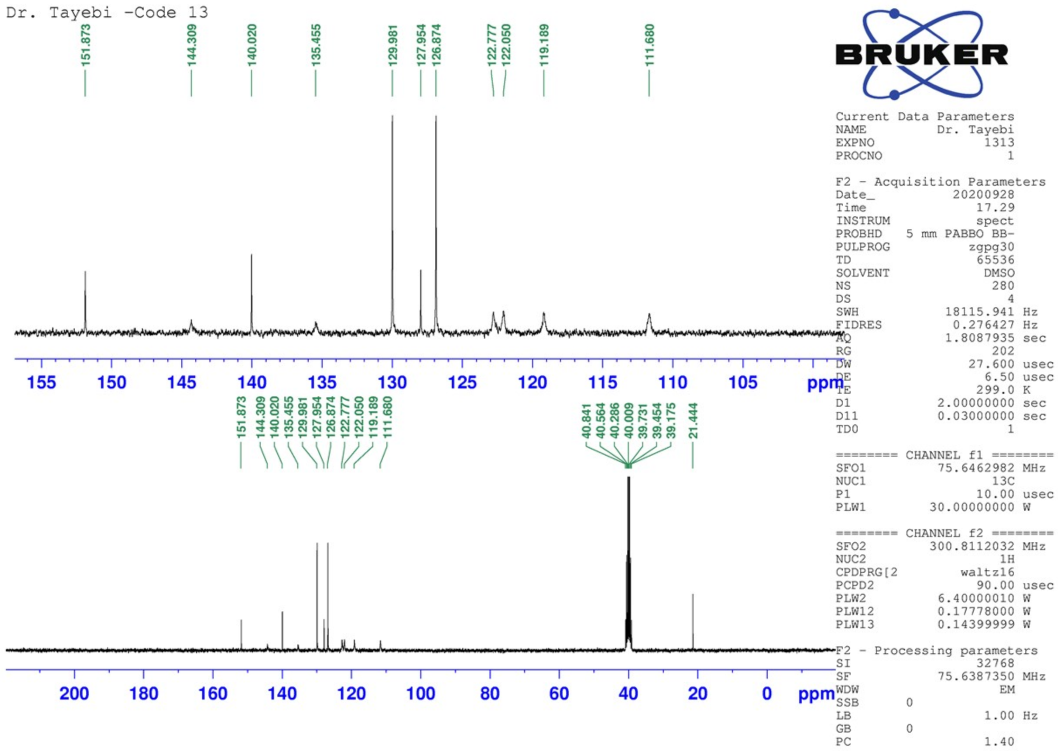

Fig. S2 ^{13}C-NMR Spectrum of 2-(4-methyl-phenyl)-benzimidazole (Table 2, entry 1)

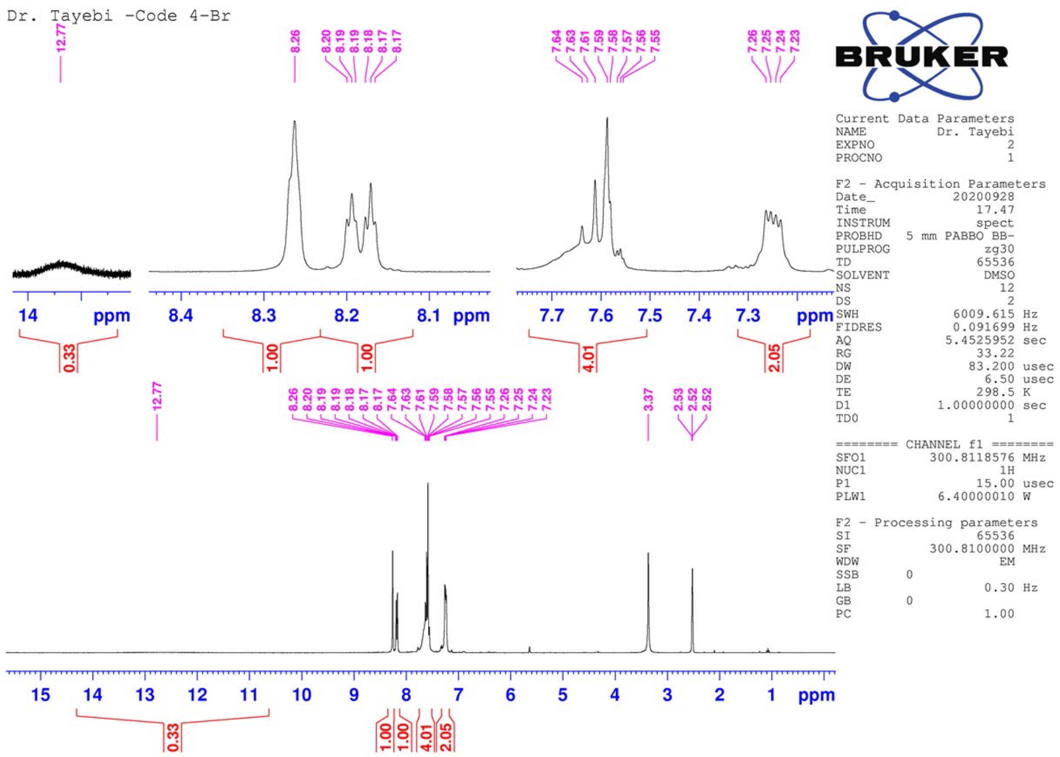

Fig. S3 ^{1}H-NMR Spectrum of 2-(4-bromo-phenyl)-benzimidazole (Table 2, entry 2)

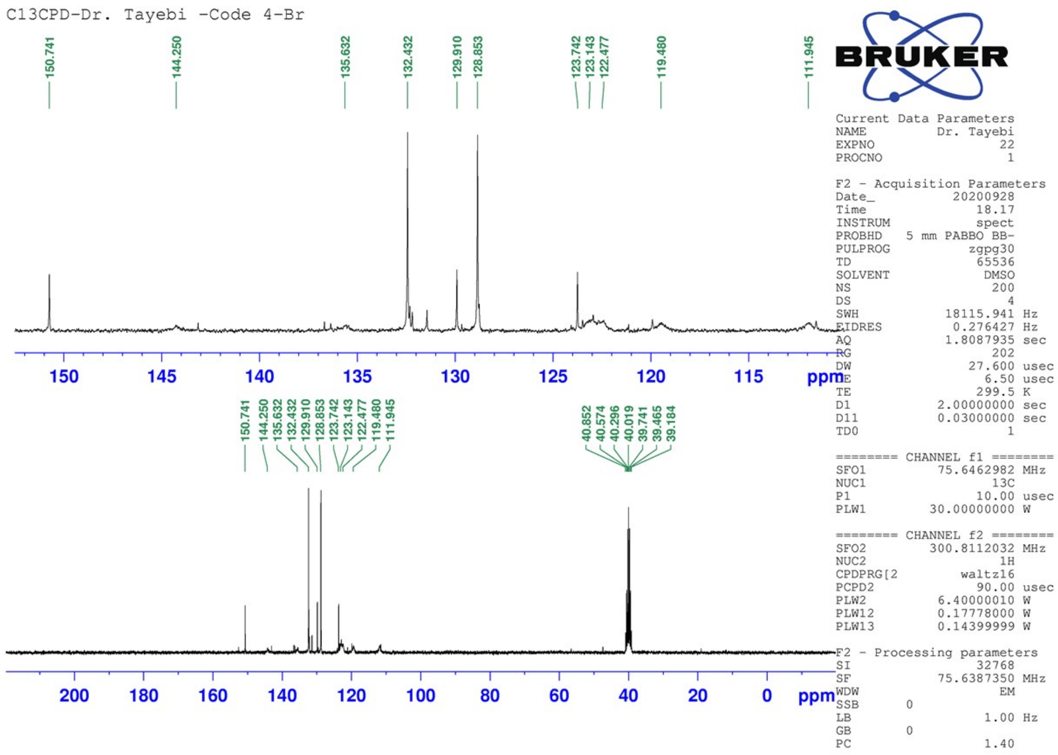

Fig. S4 ^{13}C-NMR Spectrum of 2-(4-bromo-phenyl)-benzimidazole (Table 2, entry 2)

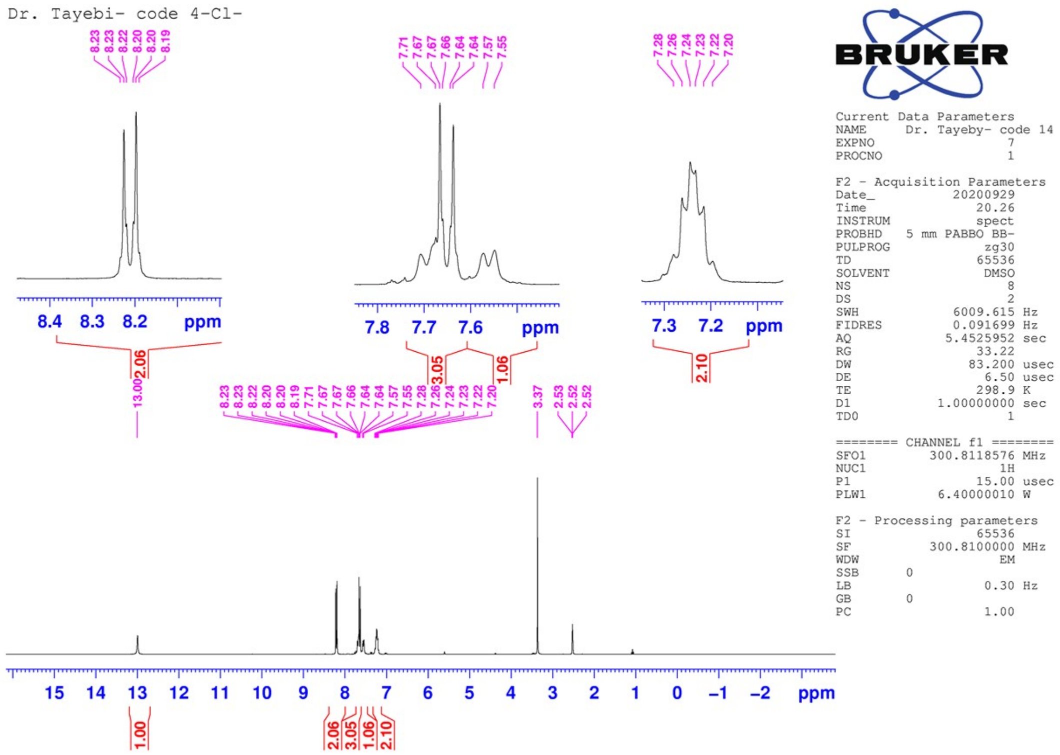

Fig. S5 ^{1}H-NMR Spectrum of 2-(4-chloro-phenyl)-benzimidazole (Table 2, entry 3)

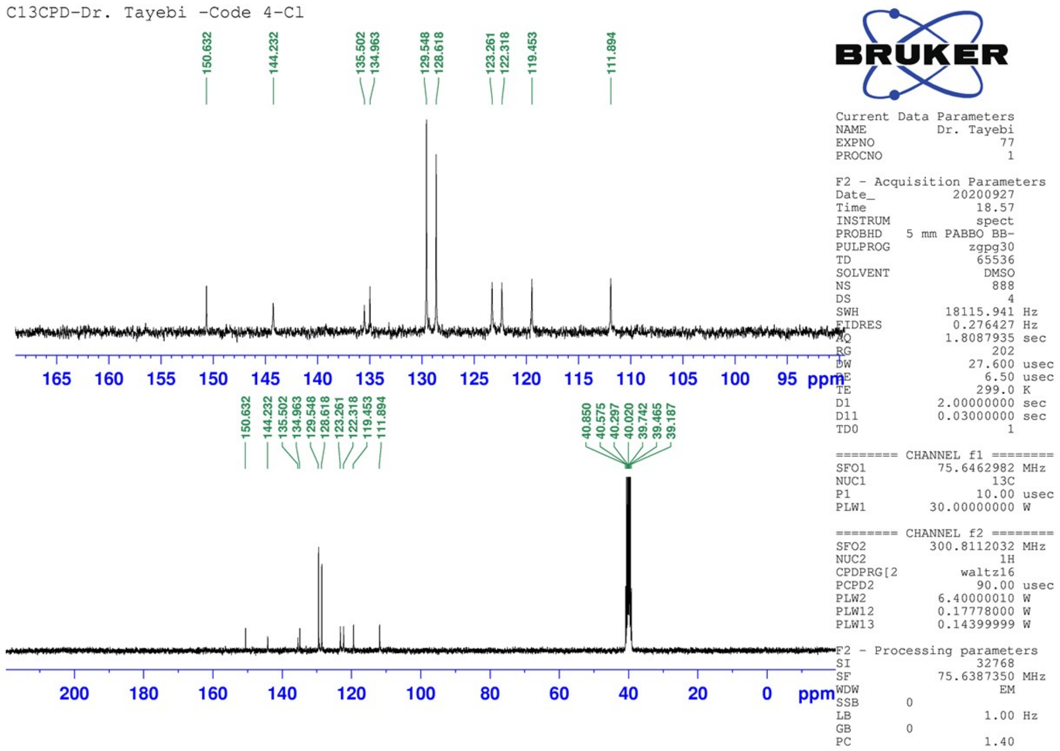

Fig. S6 ^{13}C-NMR Spectrum of 2-(4-chloro-phenyl)-benzimidazole (Table 2, entry 3)

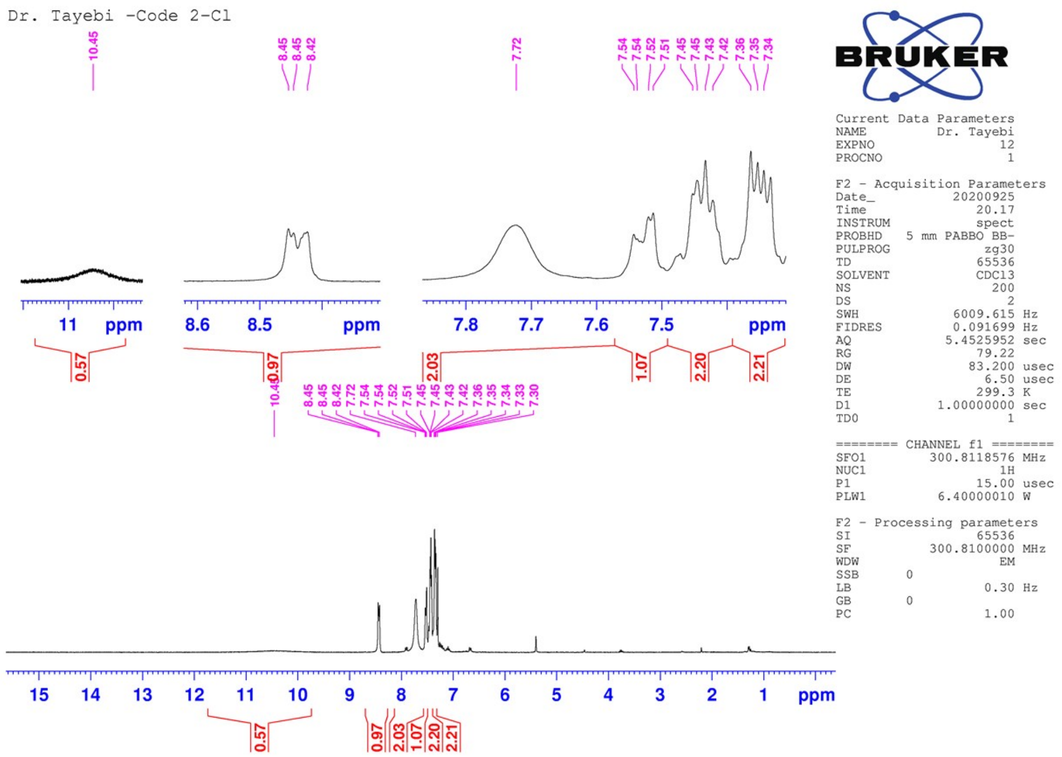

Fig. S7 ^{1}H-NMR Spectrum of 2-(2-chloro-phenyl)-benzimidazole (Table 2, entry 4)

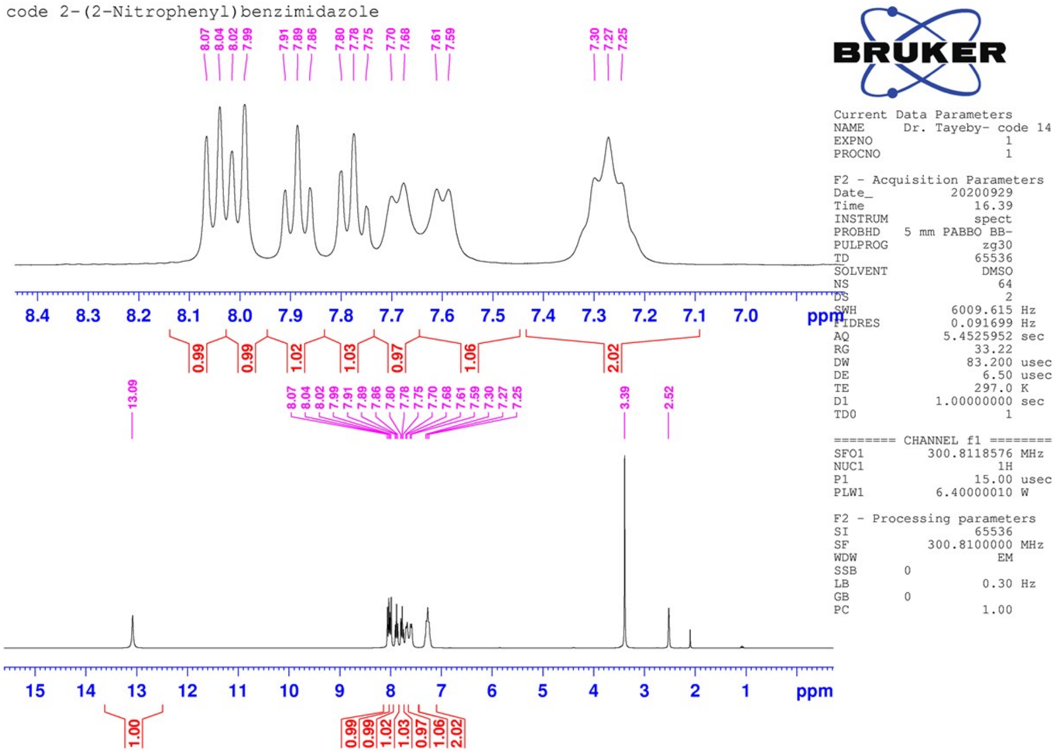

Fig. S8 ^{1}H-NMR Spectrum of 2-(2-nitro-phenyl)-benzimidazole (Table 2, entry 5)

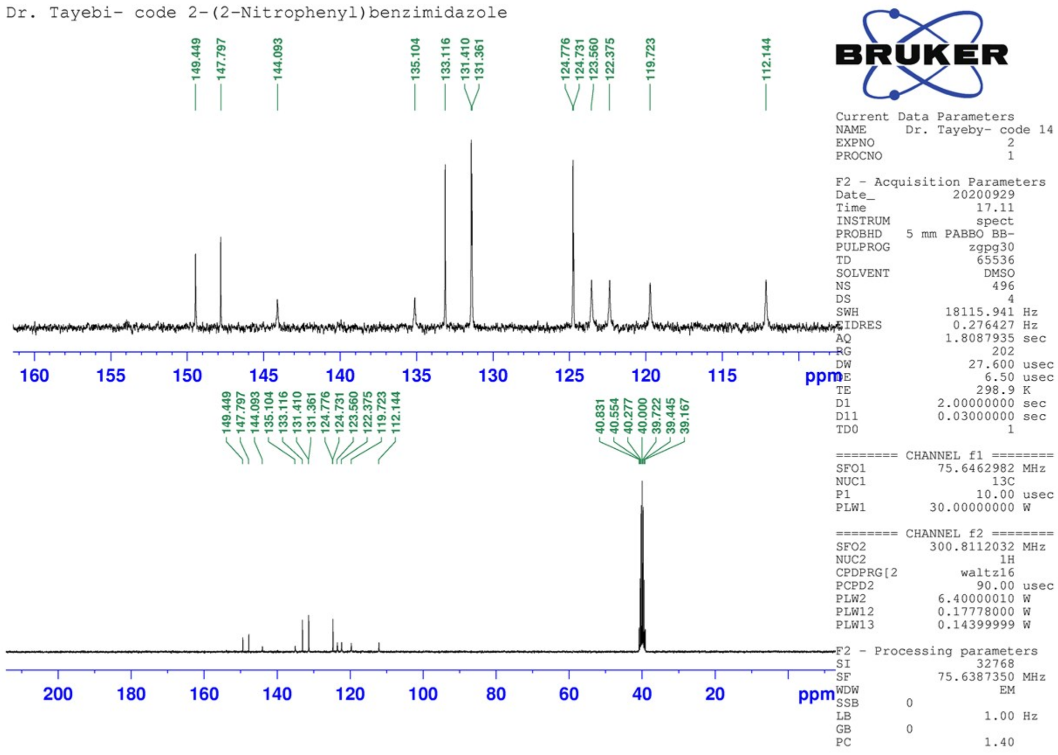

Fig. S9 ^{13}C-NMR Spectrum of 2-(2-nitro-phenyl)-benzimidazole (Table 2, entry 5)

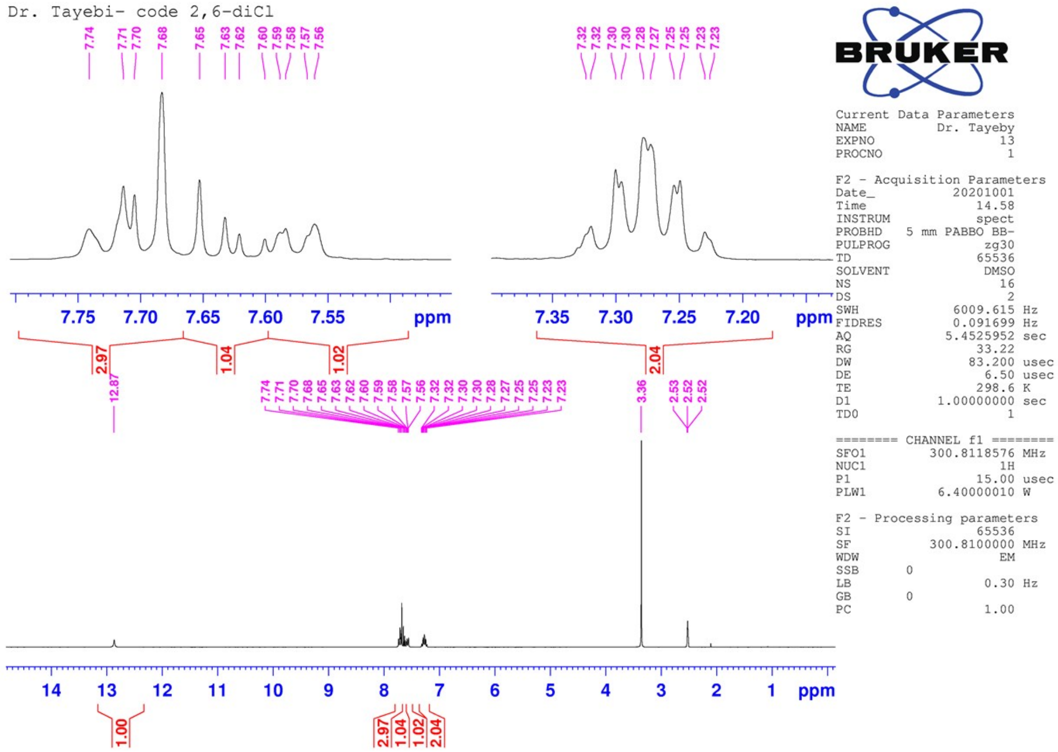

Fig. S10 ^{1}H-NMR Spectrum of 2-(2, 6-dichloro-phenyl)-benzimidazole (Table 2, entry 6)

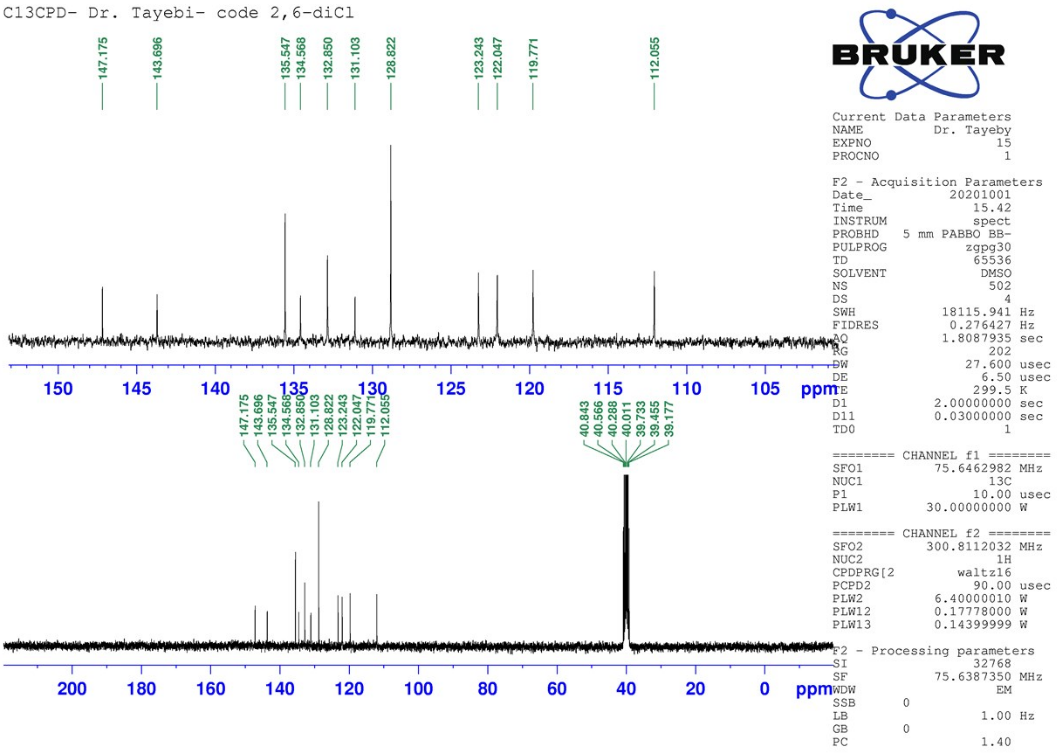

Fig. S11 ^{13}C-NMR Spectrum of 2-(2, 6-dichloro-phenyl)-benzimidazole (Table 2, entry 6)

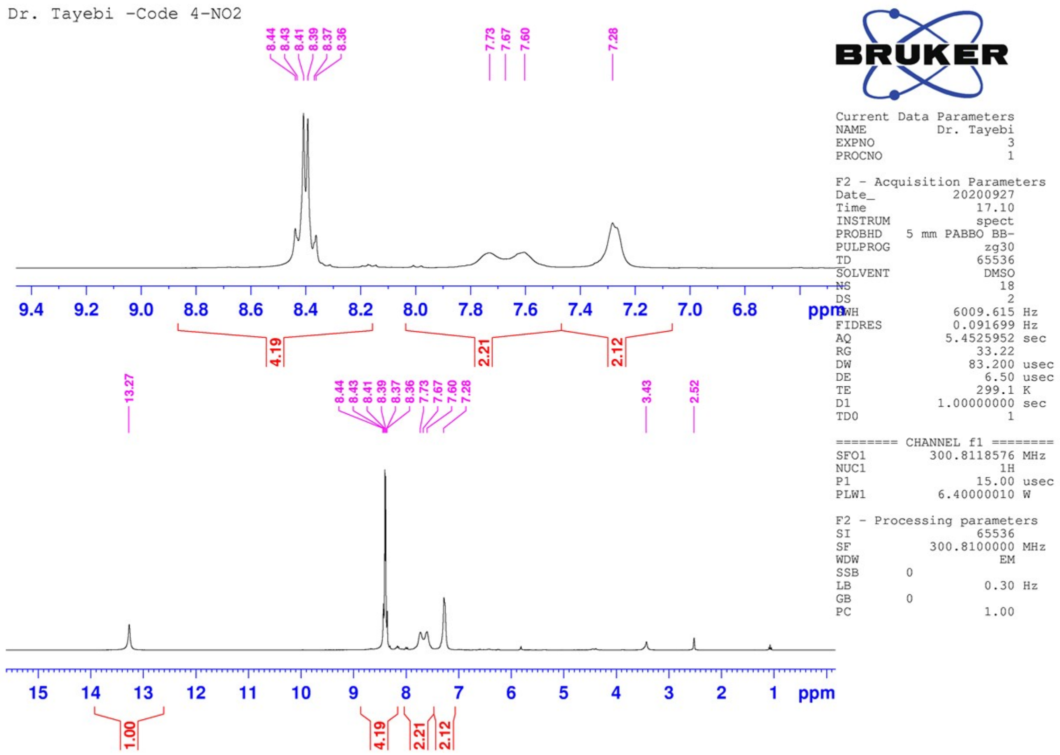

Fig. S12 ^{1}H-NMR Spectrum of 2-(4-nitro-phenyl)- benzimidazole (Table 2, entry 7)

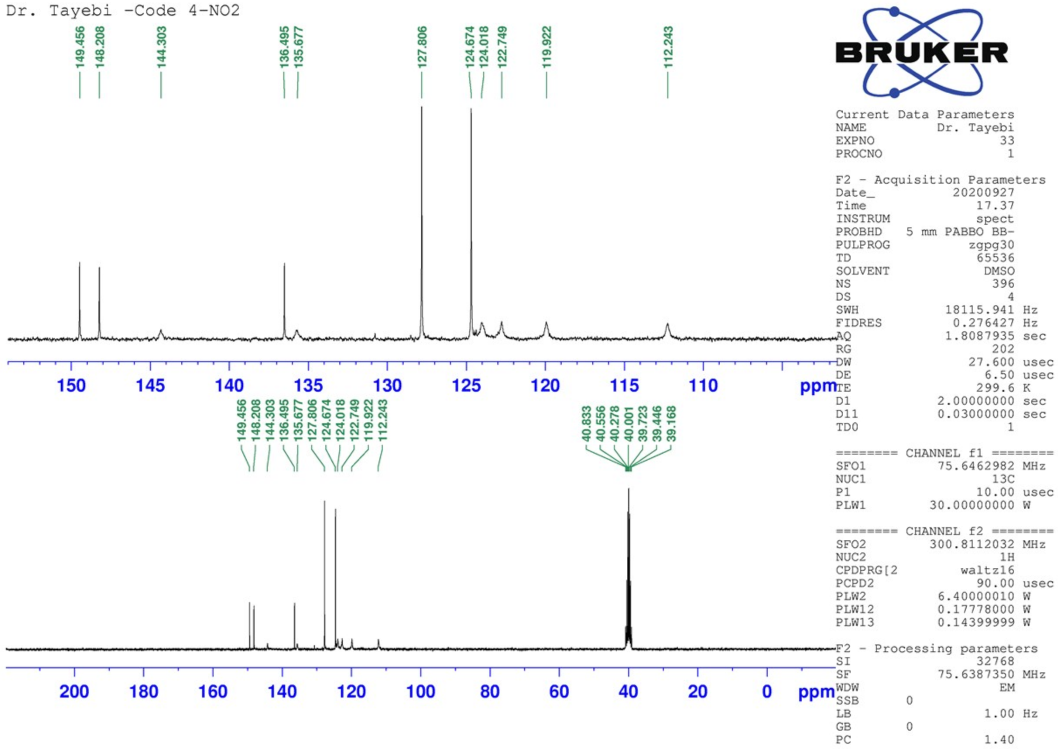

Fig. S13 ^{13}C-NMR Spectrum of 2-(4-nitro-phenyl)- benzimidazole (Table 2, entry 7)

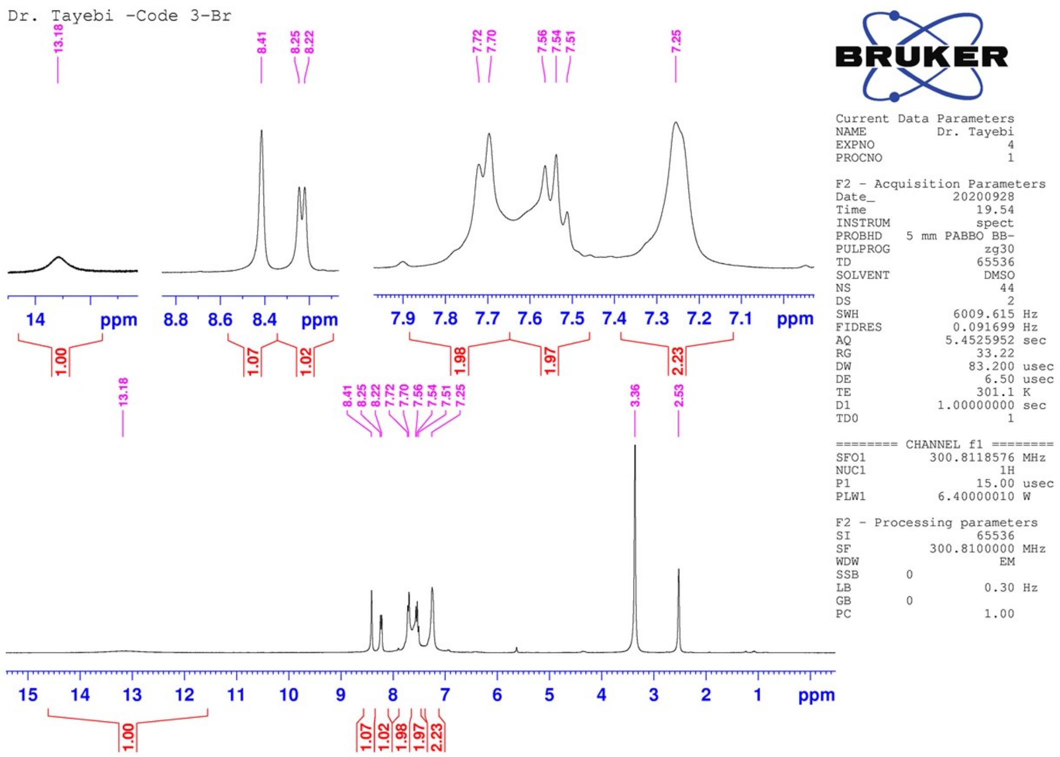

Fig. S14 ^{1}H-NMR Spectrum of 2-(3-bromo-phenyl)-benzimidazole (Table 2, entry 8)

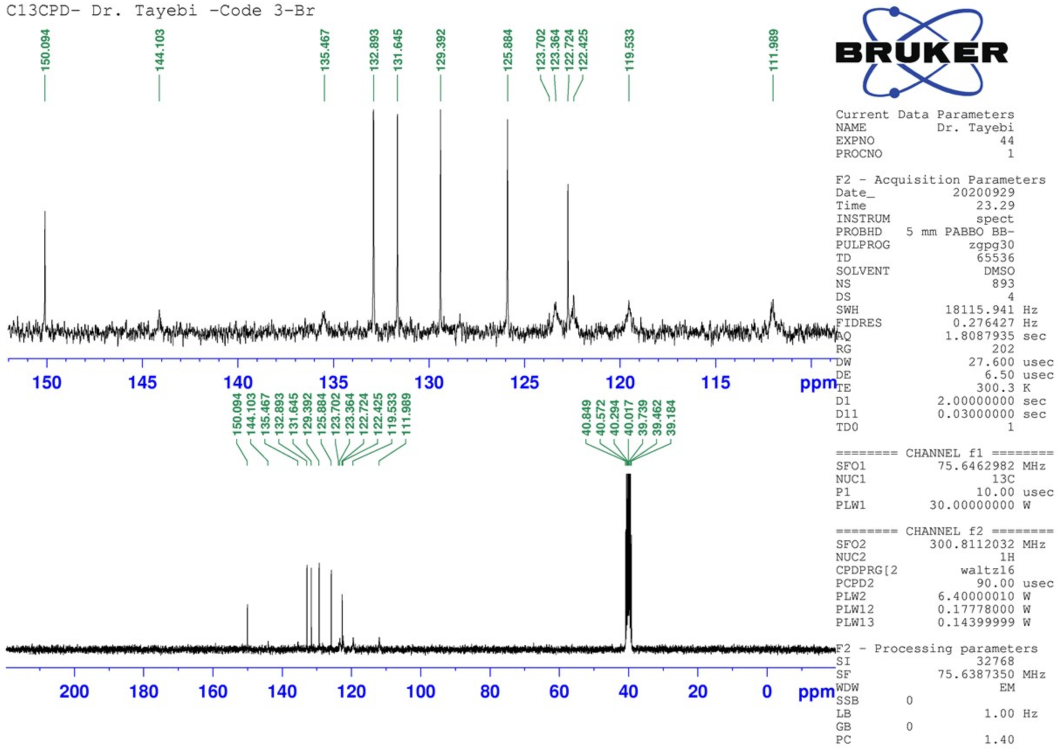

Fig. S15 ^{13}C-NMR Spectrum of 2-(3-bromo -phenyl)-benzimidazole (Table 2, entry 8)

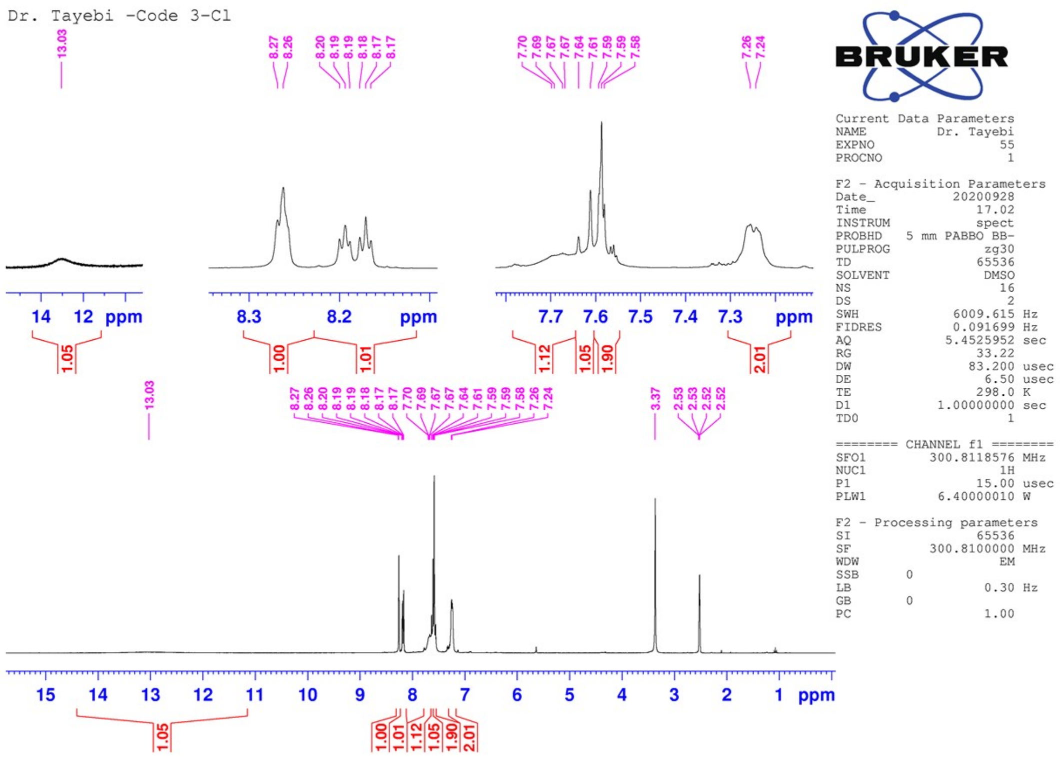

Fig. S16 ^{1}H-NMR Spectrum of 2-(3-chloro-phenyl)-benzimidazole (Table 2, entry 9)

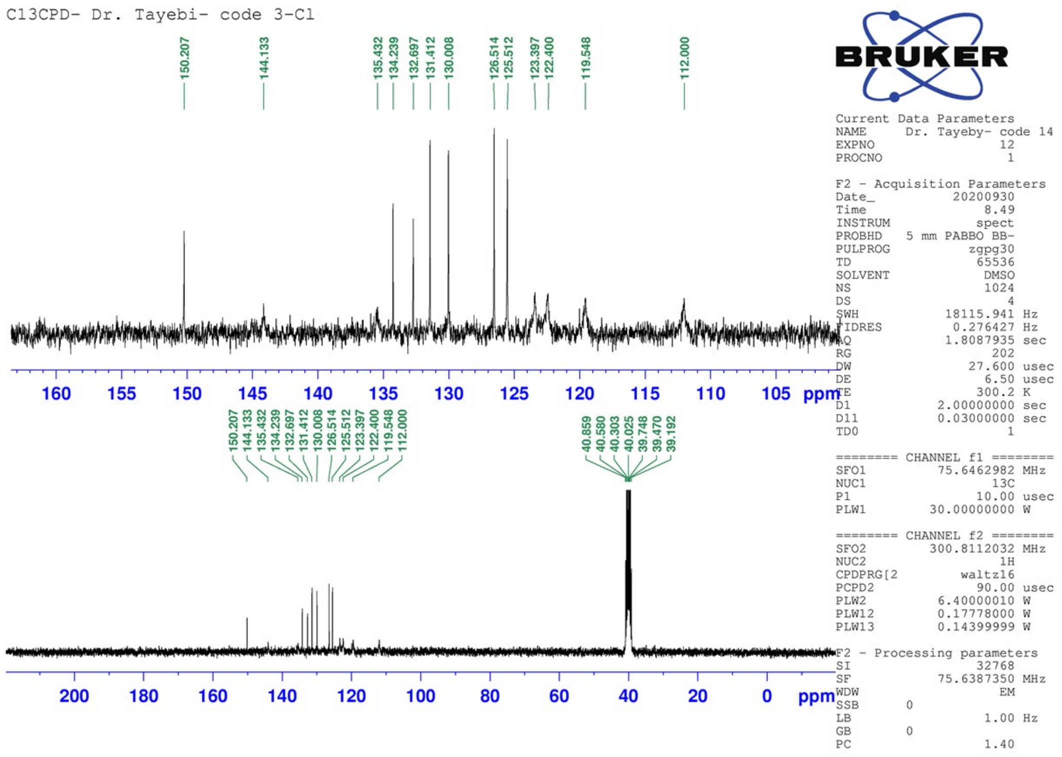

Fig. S17 ^{13}C-NMR Spectrum of 2-(3-chloro-phenyl)-benzimidazole (Table 2, entry 9)

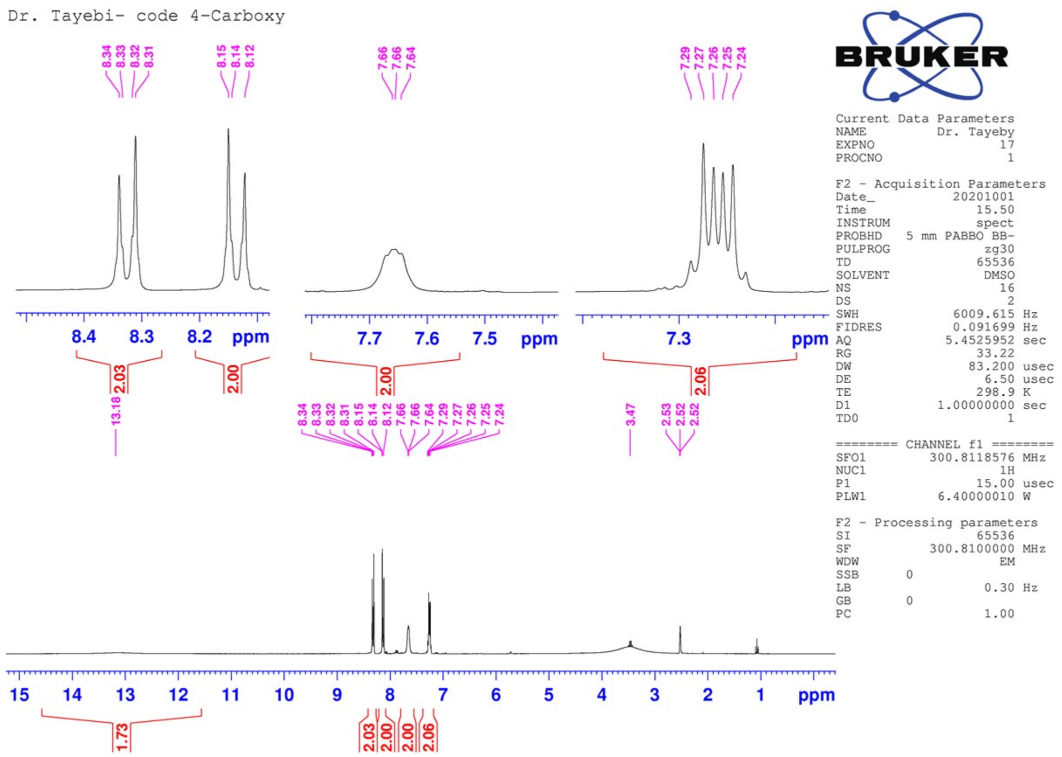

Fig. S18 ^{1}H-NMR Spectrum of 2-(4-carboxy-phenyl)-benzimidazole (Table 2, entry 10)

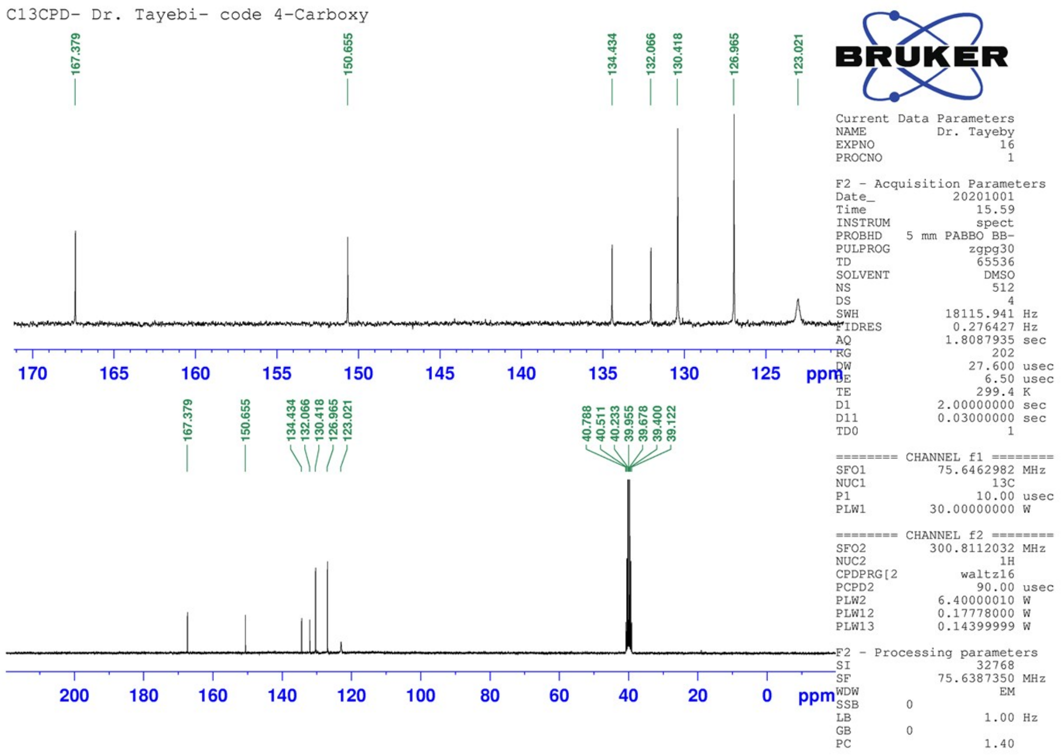

Fig. S19 ^{13}C-NMR Spectrum of 2-(4-carboxy-phenyl)-benzimidazole (Table 2, entry 10)

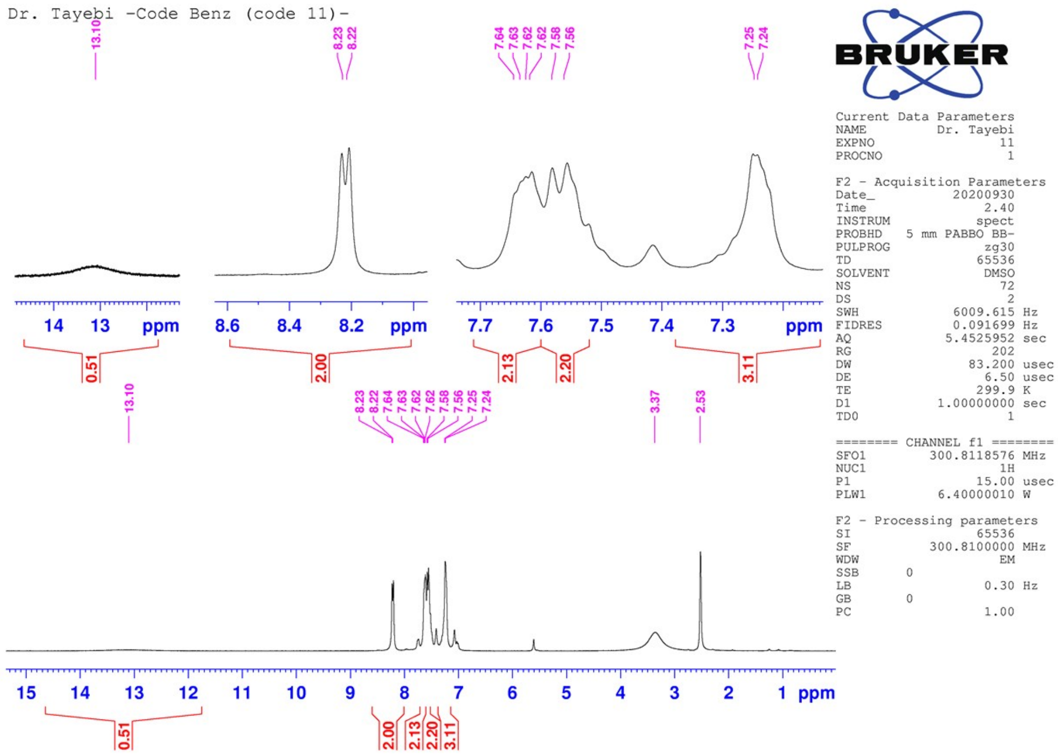

Fig. S20. ^{1}H-NMR Spectrum of 2-(phenyl)-benzimidazole (Table 2, entry 11)

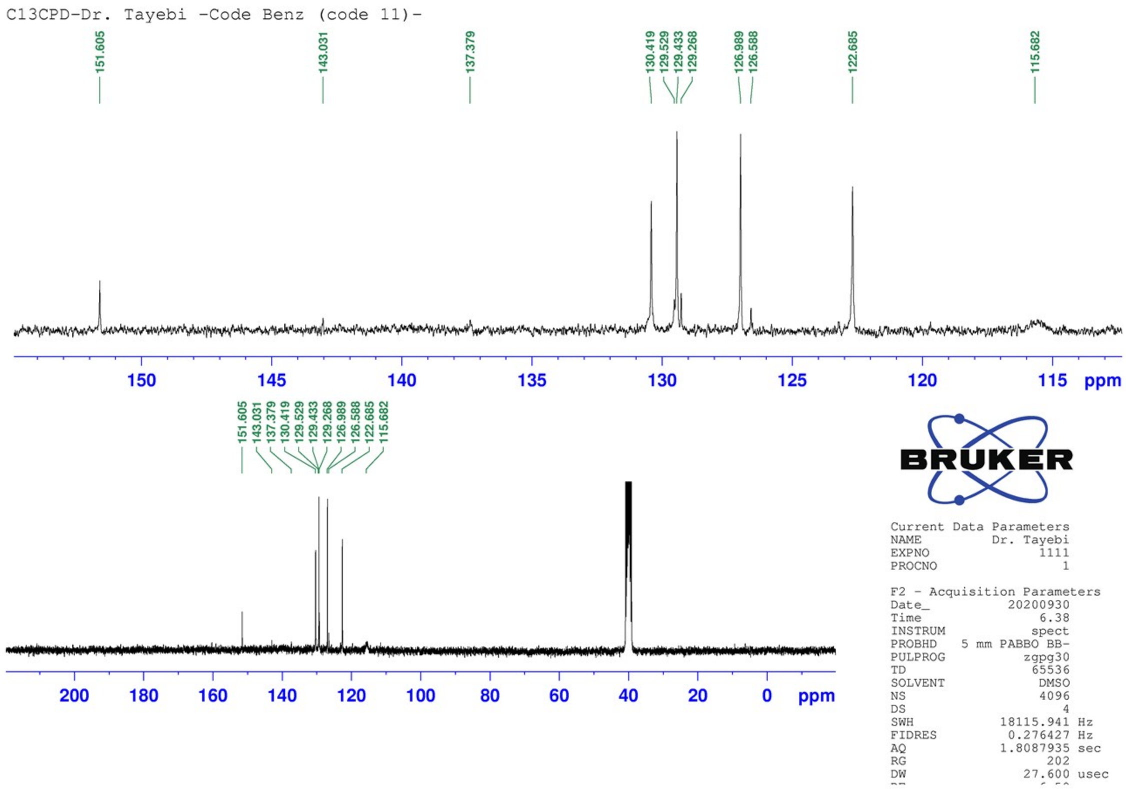

Fig. S21. ^{13}C-NMR Spectrum of 2-(phenyl)-benzimidazole (Table 2, entry 11)

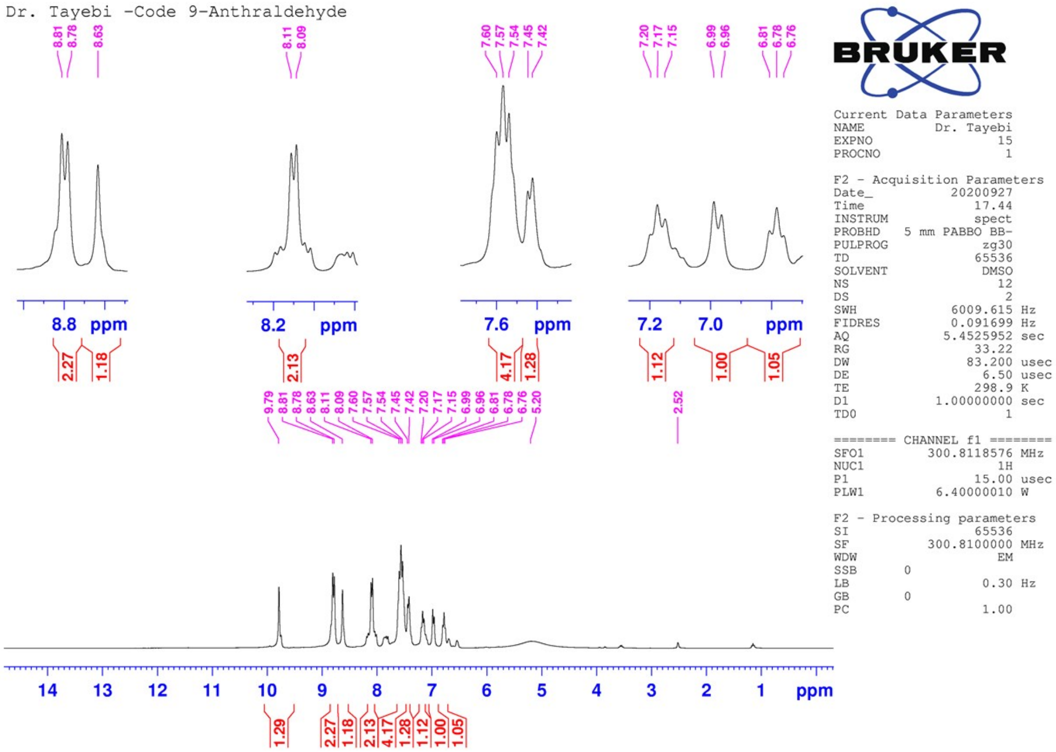

Fig. S22. ^{1}H-NMR Spectrumof 2-(9-anthracene)-benzimidazole (Table 2, entry 12)

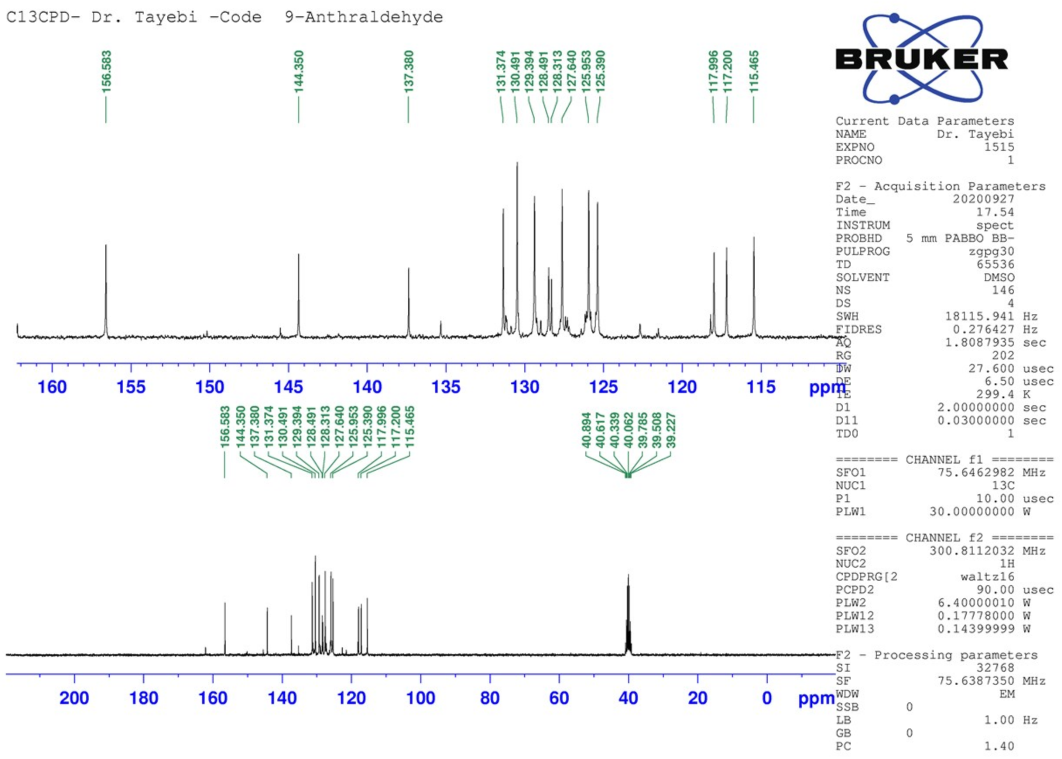

Fig. S23 ^{13}C-NMR Spectrum of 2-(9-anthracene)-benzimidazole (Table 2, entry 12)

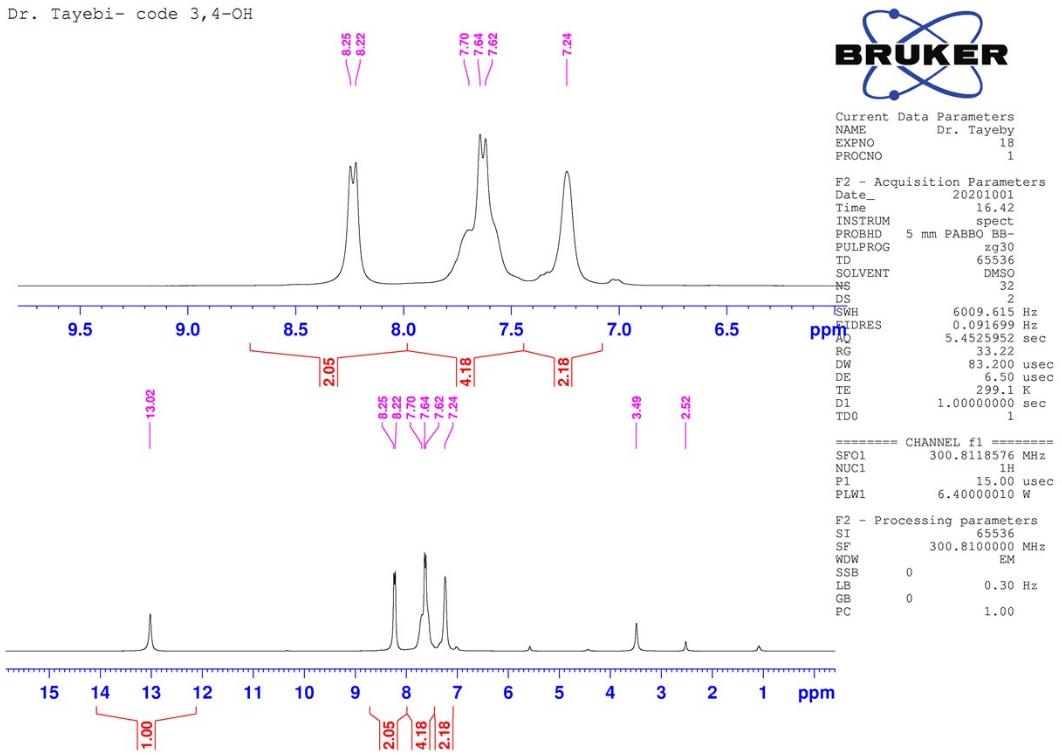

Fig. S24 ^{1}H-NMR Spectrum of 2-(3,4-dihydroxy-phenyl)-benzimidazole (Table 2, entry 13)

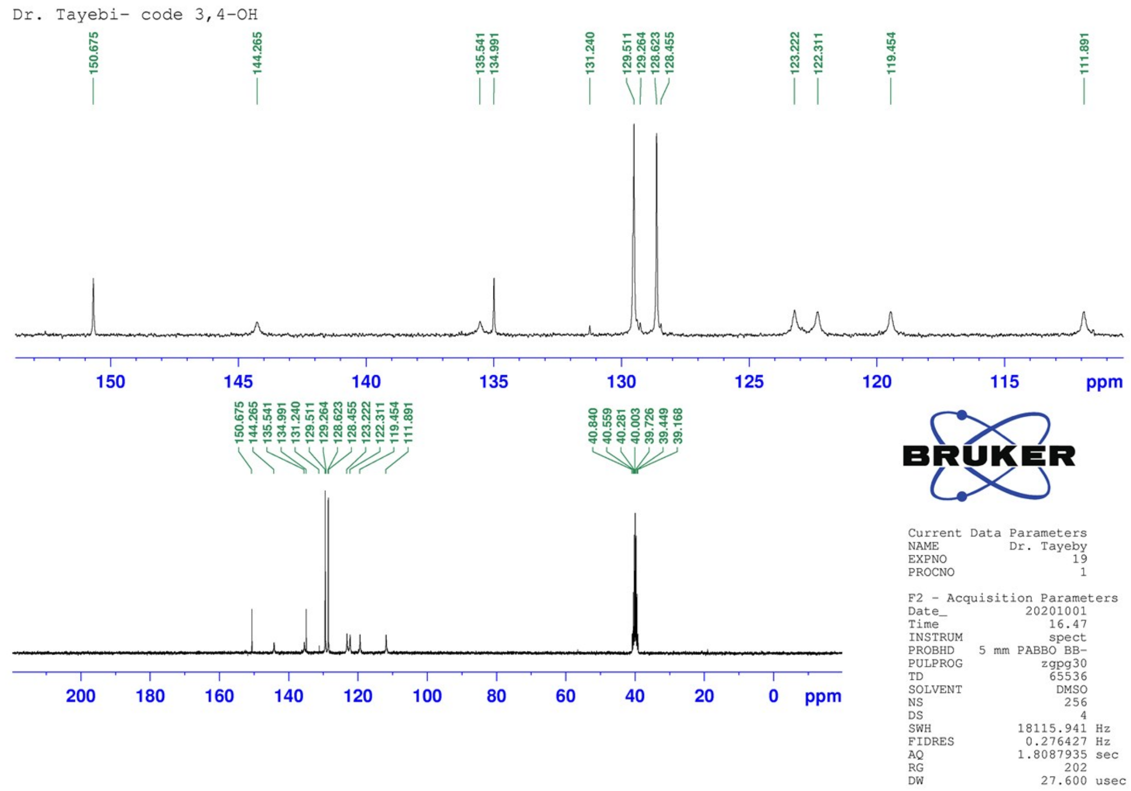

Fig. S25 ^{13}C-NMR Spectrum of 2-(3,4-dihydroxy-phenyl)-benzimidazole (Table 2, entry 13)
